# Ruthenium-Based Catalytic Systems Incorporating a Labile Cyclooctadiene Ligand with *N*-Heterocyclic Carbene Precursors for the Atom-Economic Alcohol Amidation Using Amines

**DOI:** 10.3390/molecules23102413

**Published:** 2018-09-20

**Authors:** Cheng Chen, Yang Miao, Kimmy De Winter, Hua-Jing Wang, Patrick Demeyere, Ye Yuan, Francis Verpoort

**Affiliations:** 1 State Key Laboratory of Advanced Technology for Materials Synthesis and Processing, Wuhan University of Technology, 122 Luoshi Road, Wuhan 430070, China; chengchen@whut.edu.cn; 2 School of Materials Science and Engineering, Wuhan University of Technology, 122 Luoshi Road, Wuhan 430070, China; miaoyang2018@126.com; 3 Odisee/KU Leuven Technology Campus, Gebroeders de Smetstraat 1, 9000 Ghent, Belgium; kimmydewinter@hotmail.com (K.D.W.); patrick.demeyere@odisee.be (P.D.); 4 School of Chemistry, Chemical Engineering and Life Sciences, Wuhan University of Technology, 122 Luoshi Road, Wuhan 430070, China; 18772101949@163.com; 5 National Research Tomsk Polytechnic University, Lenin Avenue 30, Tomsk 634050, Russian; 6 Ghent University Global Campus, 119 Songdomunhwa-Ro, Yeonsu-Gu, Incheon 21985, Korea

**Keywords:** ruthenium (Ru), *N*-heterocyclic carbenes (NHCs), homogeneous catalysis, in situ, amide bonds, synthesis

## Abstract

Transition-metal-catalyzed amide-bond formation from alcohols and amines is an atom-economic and eco-friendly route. Herein, we identified a highly active in situ *N*-heterocyclic carbene (NHC)/ruthenium (Ru) catalytic system for this amide synthesis. Various substrates, including sterically hindered ones, could be directly transformed into the corresponding amides with the catalyst loading as low as 0.25 mol.%. In this system, we replaced the *p*-cymene ligand of the Ru source with a relatively labile cyclooctadiene (cod) ligand so as to more efficiently obtain the corresponding poly-carbene Ru species. Expectedly, the weaker cod ligand could be more easily substituted with multiple mono-NHC ligands. Further high-resolution mass spectrometry (HRMS) analyses revealed that two tetra-carbene complexes were probably generated from the in situ catalytic system.

## 1. Introduction

Amides are a series of fundamental functional structures in nature and biological systems, as well as crucial building blocks for organic synthesis [1,2,3,4,5,6]. As of late, numerous synthetic methods were reported for the construction of amide bonds. However, they generally suffer from the usage of various stoichiometric additives and the production of unfavorable equimolar byproducts [7,8,9,10,11,12,13,14]. Therefore, green and eco-friendly strategies are highly required for amide synthesis [15]. Recently, a methodology employing transition-metal-based catalytic systems for direct amide synthesis from alcohols and amines was proven to be far more atom-economic and environmentally friendly as the only byproduct is hydrogen [16,17,18,19,20,21,22]. Throughout this research, ruthenium (Ru) was most extensively studied [23]. Initially, the Murahashi [24] and Milstein [25] groups pioneered Ru-catalyzed amide synthesis in intramolecular and intermolecular manners, respectively. Notably, the Milstein catalyst, a Ru complex bearing a PNN-type pincer ligand, was highly active for this reaction. With a catalyst loading of 0.1 mol.%, various amides could be synthesized from alcohols and amines [25]. Later, great progress was achieved by the Milstein [26,27,28], Madsen [29,30,31], Williams [32,33], Hong [34,35,36,37,38,39,40,41,42,43], Crabtree [44,45], Albrecht [46], Guan [47,48], Glorius [49], Möller [50,51], Bera [52], Huynh [53], Viswanathamurthi [54,55,56], Mashima [57], Verpoort [58,59], and Kundu [60] groups. In particular, Ru combined with *N*-heterocyclic carbenes (NHCs) attracted more and more interest due to the flexible tunability of the electronic and steric properties of NHCs, which may easily access the optimal structures of the corresponding NHC/Ru complexes [61,62,63]. Accordingly, a multitude of efficient NHC/Ru catalytic systems were discovered for this reaction. Furthermore, considering the merits of the in situ catalytic systems, such as easy operation and convenient investigation of electronically and sterically distinct NHCs, a number of versatile and potent in situ NHC/Ru catalytic systems recently emerged. However, satisfactory yields could only be attained by these reported systems if relatively high Ru loadings of 2.0–5.0 mol.% were employed [29,34,36,37,49]. Therefore, the development of more efficient in situ NHC/Ru catalytic systems which can accomplish the formation of amide linkage are urgently required.

**Figure 1:**
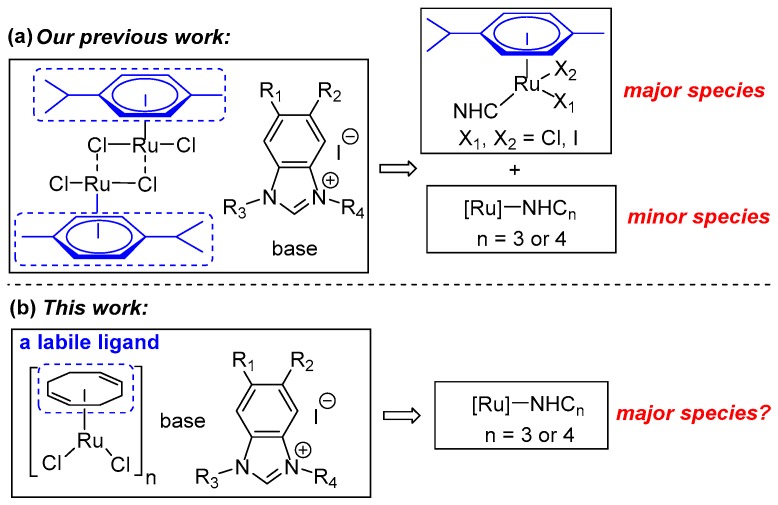
The design strategy of this work.

In our previous work, the development of various in situ generated (*p*-cymene)/Ru catalytic systems, which contain benzimidazole-based NHC precursors bearing different electronic and steric properties, was accomplished [58]. Further experiments revealed that two mono-NHC/Ru complexes were observed as major species and two poly-carbene complexes were detected as only minor species (as depicted in Figure 1a) [59]. Herein, we envisioned that replacing the *p*-cymene ligand of the Ru center with a relatively labile cyclooctadiene (cod) ligand could possibly give rise to poly-carbene complexes as a major species (as shown in Figure 1b). Expectedly, the weaker cod ligand could be more easily substituted with multiple mono-NHC ligands. Based on this, an efficient in situ NHC/Ru catalytic system was developed through extensive screening of various conditions. Notably, this system demonstrated excellent catalytic activity for amide synthesis with the applied catalyst loading as low as 0.25 mol.%. Various amides, including sterically congested ones, were directly synthesized from alcohols and amines in moderate to excellent yields. Furthermore, high-resolution mass spectrometry (HRMS) analyses suggested several Ru species bearing multiple NHC ligands as major species, which was in accordance with our prospection.

## 2. Results and Discussion

The reaction of benzyl alcohol (**1a**) and benzylamine (**2a**) was selected as a model reaction for the optimization of the reaction conditions. Based on our previous work [59], 0.5 mol.% of [RuCl_2_(cod)]_n_, 2.00 mol.% of an NHC precursor, 3.50 mol.% of NaH, 0.5 h of catalyst generation time, and 16 h of reaction time were originally applied (as listed in Table 1). In the beginning, NHC precursors **L1**–**L6** with different backbone and wingtip substituents were prepared (entries 1–6, Table 1). The first and foremost, 62% of amide **3a** and 15% of imine **4a**, were obtained with 18% of **1a** remaining if **L1** was used (entry 1). Electron-deficient precursor **L2** gave rise to lower amide content in the product distribution, demonstrating its disadvantage for amide formation (entry 2 vs. entry 1). In the case of an electron-rich NHC precursor (**L3**), a similar result was obtained compared with **L1** (entry 3 vs. entry 1). Moreover, the substituents on the *N*-terminus of the NHC precursors were adjusted (entries 1, 4–6). With retaining Me as the substituent for one *N*-terminus, different groups including Et, *n*Pr, and *i*Pr were introduced for the other terminus. The result was indicative that Et was the optimized group for this reaction (entry 4 vs. entries 1, 5, and 6). After establishing the ideal NHC precursor (**L4**), we continued the optimization by screening other reaction conditions. It was found that the catalyst generation time was crucial for the catalysis (entries 4, 7–11); 57% of the amide product could be detected if every substance was added simultaneously (entry 5). As we elongated the period for the in situ catalyst generation from 0 h to 2.0 h, the yields of **3a** gradually increased (entries 4, 7–10). A further increment of the time led to a similar yield (entry 11 vs. entry 10). Therefore, the ideal duration for the catalyst generation was finalized as 2 h. Next, the ratio of [Ru]: **L4**:NaH was varied (entries 12–17). It is worth emphasizing that the amount of both **L4** and NaH changed so as to ascertain three additional equivalents of NaH to activate [RuCl_2_(cod)]_n_ for all cases. Without **L4**, no amide was formed (entry 12). As the ratio increased from 1:0:3 to 1:5:8, gradually higher yields of **3a** were observed (entries 10, 12–16). However, a higher ratio prompted a reduced yield of **3a** (entry 17 vs. entry 16). Thus, the ratio of 1:5:8 was recognized as the best one (entry 16), and further increasing the reaction time from 16 h to 36 h produced **3a** in 93% yield (entry 18).

In order to identify a more active catalytic system, a reduced Ru loading of 0.25 mol.% was attempted (as listed in Table 2). At the outset, 65% of **3a** was afforded if the loading of the above-optimized catalytic system was directly reduced to 0.25 mol.% (entry 1). In addition, different bases including potassium bis(trimethylsilyl)amide (KHMDS), KO*t*Bu, and Cs_2_CO_3_ were exploited instead of NaH (entries 2–4). Interestingly, compared with NaH, the milder Cs_2_CO_3_ led to an increased yield of **3a** (entry 4 vs. entry 1). It was also noticed that the volume of toluene was crucial for the reaction (entries 4–8). Either a more concentrated or diluted solution triggered a lower amide/imine selectivity (entry 5–8 vs. entry 4). Furthermore, the adjustment of the base amounts influenced the reaction (entries 4, 9–12), and 1.75 mol.% of Cs_2_CO_3_ was found to be optimal for the selective amide formation (entry 10). Therefore, the optimized reaction conditions were identified as **1** (5.00 mmol), **2** (5.50 mmol), [RuCl_2_(cod)]_n_ (0.0125 mmol), **L4** (0.0625 mmol), Cs_2_CO_3_ (0.075 mmol), toluene (1.50 mL), reflux, and 36 h unless otherwise noted.

**Figure 2:**
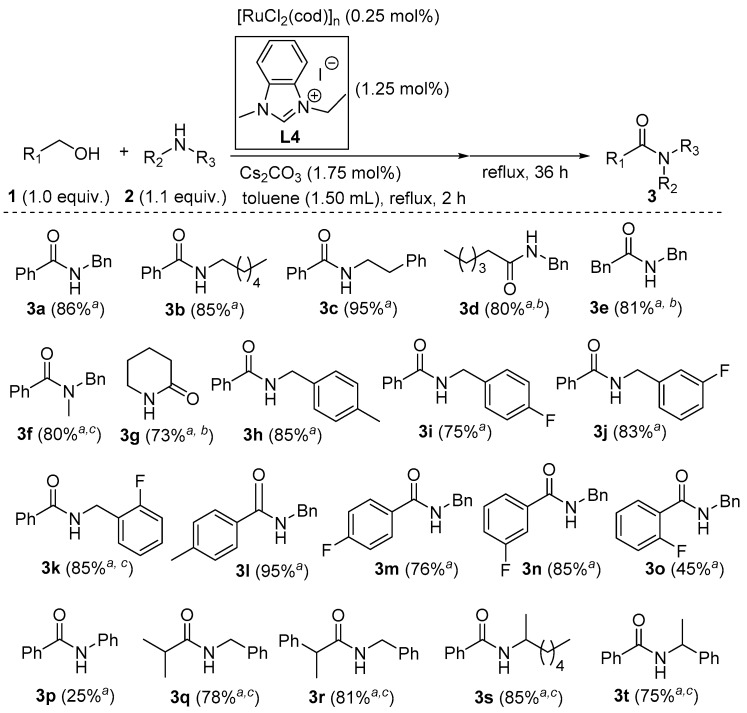
Amide synthesis from various alcohols and amines; *^a^* isolated yields (averages of two consistent runs); *^b^* in *m*-xylene at reflux; *^c^* 0.5 mol.% of [Ru].

With the optimized reaction conditions at hand, the substrate scope and limitations of this strategy were further investigated (as depicted in Figure 2). For the sterically non-hindered substrates (**1a**–**1e)**, the corresponding amides could be obtained in good to excellent yields. If a secondary amine (**1f**) was employed, tertiary amide **3f** was also given in 80% yield with 0.5 mol.% of [Ru]. Expectedly, lactam **3g** was efficiently afforded from amino alcohol **1f** in an intramolecular pattern. On the other hand, the reactions of benzyl alcohol with substituted benzylamines were evaluated. It seemed that these substituents had no obvious influence on the reactivity, and amides **3h**–**3k** were synthesized in 75–85% yields. In the case of coupling benzylamine with various benzyl alcohols, a substituent at either the *para* or *meta* position resulted in good yields of amides **3l**–**3n**. However, an *ortho* group gave amide **3o** in a moderate yield. Apparently, aromatic amines were less reactive, and aniline (**2p**) produced amide **3p** in only 25% yield. To our delight, this newly developed catalytic system was not as sensitive to steric bulks as our previous systems [58,59]. With an Ru loading of 0.5 mol.%, several sterically hindered substrates could be efficiently transformed into amides **3q**–**3t**.

**Figure 3:**
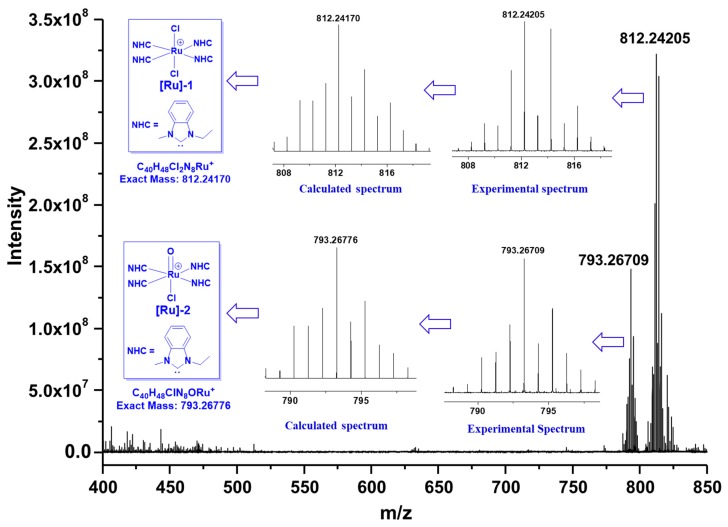
The high-resolution mass spectrometry (HRMS) analyses for the identification of the possible Ru species.

Concerning the in situ catalytic systems, it is crucial to explore the possible structures of the generated Ru species. As a result, HRMS analyses were performed to clarify this matter (as shown in Figure 3). In accordance with our speculation, no mono-carbene complexes were detected. Instead, two poly-carbene Ru species were observed from the spectrum. [**Ru**]-**1** (corresponding to an isotopic peak at *m*/*z* = 812.24209), consistent with an Ru species comprising four-fold NHC ligands, was observed as a major species. Furthermore, another tetra-carbene Ru species, assigned as **[Ru]**-**2** with the isotopic peak at *m*/*z* = 793.26709, was also found as a minor species. Presumably, during exposure to air and/or the HRMS measurements, the Ru centers in **[Ru]**-**1** and **[Ru]**-**2** were oxidized to +3 and +4, respectively. Unfortunately, attempts to isolate these tetra-carbene complexes were unsuccessful, probably due to the complexity of the in situ catalyst generation. Therefore, it was still unclear whether the high activity of the current catalytic system was attributed to the observed tetra-carbene Ru species or other species.

## 3. Experimental

### 3.1. General Considerations

All reactions were carried out using standard Schlenk techniques or in an argon-filled glove box unless otherwise mentioned. All the substrates and solvents were obtained from commercial suppliers and used as received without further purification. ^1^H-NMR spectra were recorded on a Bruker Avance 500 spectrometer (Billerica, MA, USA) in CDCl_3_ or DMSO-*d*_6_ with TMS as the internal reference, and ^13^C-NMR spectra were recorded in CDCl_3_ or DMSO-*d*_6_ on a Bruker Avance 500 (126 MHz) spectrometer. The following abbreviations were used to designate multiplicities: s = singlet, brs = broad singlet, d = doublet, t = triplet, dd = doublet of doublets, dq = doublet of quartets, td = triplet of doublets, ddd = doublet of doublets of doublets, and m = multiplet. Melting points were taken on a Buchi M-560 melting point apparatus (Flawil, Switzerland) and were uncorrected. HRMS analyses were done with a Bruker Daltonics microTOF-QII instrument (Billerica, MA, USA). NHC precursors **L1**–**L6** were prepared according to a previous publication [58,59], and all the amide products were identified by spectral comparison with the literature data [58,59]. ^1^H-NMR, ^13^C-NMR data and original spectra of amides **3a**–**3t** could be found in the Supplementary Materials.

### 3.2. General Procedure for the Amide Synthesis

Inside an argon-filled glove box, [Ru(cod)Cl_2_]_n_ (3.5 mg, 0.0125 mmol), **L4** (18.0 mg, 0.0625 mmol), Cs_2_CO_3_ (28.6 mg, 0.0875 mmol), and dry toluene (1.50 mL) were added to an oven-dried 25-mL Schlenk flask. The tube was taken out of the glove box and heated to reflux under argon for 2 h. Then, an alcohol (5.00 mmol) and an amine (5.50 mmol) were added, and the mixture was stirred at a refluxing temperature for 36 h. The procedures for calculating the NMR yields were as follows: when the reaction was complete, 1,3,5-trimethoxybenzene (0.5 mmol, 84.0 mg) and CHCl_3_ (1.0 mL) were added to the reaction mixture. Afterward, to an NMR tube was added 0.1 mL of the above solution and 0.4 mL of CDCl_3_. The NMR yields were obtained based on the exact amount of 1,3,5-trimethoxybenzene. In order to obtain the isolated yields of the amides, the reaction mixture was cooled down to room temperature, and the solvent was removed under reduced pressure. Finally, the residue was purified by silica-gel flash column chromatography to afford the amides.

## 4. Conclusions

In summary, based on the assumption that the relatively labile cod ligand could be replaced by multiple NHC ligands to obtain versatile and active catalytic systems, we prepared several NHC precursors with distinct electronic and steric properties, then combined them with [RuCl_2_(cod)]_n_ and a mild Cs_2_CO_3_ to obtain a series of in situ NHC/Ru catalytic systems. Through extensive screening of these systems and other conditions, the **L4**-based NHC/Ru catalytic system exhibited optimal activity for the dehydrogenative amidation of alcohols and amines. Various amides, especially sterically hindered ones, could be afforded in an efficient manner. Notably, the applied catalyst loading was as low as 0.25 mol.%. Further experiments revealed that the higher amount of **L4** compared to Ru probably facilitated the formation of two tetra-carbene species (**[Ru]**-**1** and **[Ru]**-**2**), which were observed from HRMS analyses. However, since the in situ catalytic system was relatively complicated, it is still uncertain whether these tetra-carbene Ru species or other species were key catalytic intermediates for this reaction.

## Figures and Tables

**Table 1:**
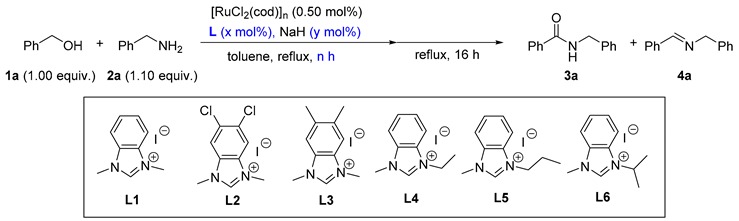
Optimization of reaction conditions with a catalyst loading of 0.5 mol.% *^a^*.

Entry	L	x	y	n	Yields (%) *^b^*		
					3a	4a	Unreacted 1a
1	**L1**	2.00	3.50	0.5	62	15	18
2	**L2**	2.00	3.50	0.5	28	30	39
3	**L3**	2.00	3.50	0.5	63	15	16
4	**L4**	2.00	3.50	0.5	78	10	8
5	**L5**	2.00	3.50	0.5	72	12	8
6	**L6**	2.00	3.50	0.5	28	30	39
7	**L4**	2.00	3.50	0.0	57	10	31
8	**L4**	2.00	3.50	1.0	79	6	8
9	**L4**	2.00	3.50	1.5	81	5	7
10	**L4**	2.00	3.50	2.0	83	4	5
11	**L4**	2.00	3.50	2.5	82	4	6
12	**L4**	0.00	1.50	2.0	0	19	76
13	**L4**	0.50	2.00	2.0	37	10	51
14	**L4**	1.00	2.50	2.0	60	11	28
15	**L4**	1.50	3.00	2.0	75	7	16
16	**L4**	2.50	4.00	2.0	86	4	9
17	**L4**	3.00	4.50	2.0	81	6	3
18 *^c^*	**L4**	**2.50**	**4.00**	**2.0**	**93**	**5**	**0**

*^a^***1a** (2.50 mmol), **2a** (2.75 mmol), [RuCl_2_(cod)]_n_ (0.50 mol.%), **L** (x mol.%), NaH (y mol.%), toluene (1.25 mL), 120 °C, n h of catalyst generation time, and 16 h of reaction time; *^b^* NMR yields (average of two consistent runs) using 1,3,5-trimethoxybenzene as an internal standard; *^c^* 36 h of reaction time.

**Table 2:**
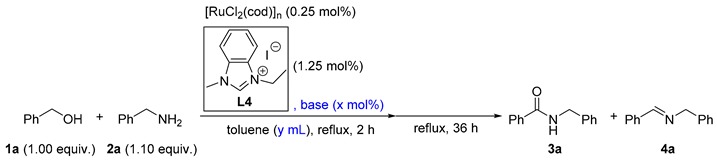
Optimization of reaction conditions with a catalyst loading of 0.25 mol.% *^a^*.

Entry	Base	x	y	Yields (%) *^b^*		
				3a	4a	Unreacted 1a
1	NaH	2.00	1.50	65	7	24
2	KHMDS	2.00	1.50	27	11	57
3	KO*t*Bu	2.00	1.50	45	15	32
4	Cs_2_CO_3_	2.00	1.50	86	7	5
5	Cs_2_CO_3_	2.00	0.50	57	18	22
6	Cs_2_CO_3_	2.00	1.00	71	13	12
7	Cs_2_CO_3_	2.00	2.00	69	16	13
8	Cs_2_CO_3_	2.00	2.50	45	38	15
9	Cs_2_CO_3_	1.50	1.50	66	15	12
**10**	**Cs_2_CO_3_**	**1.75**	**1.50**	**90**	**7**	**2**
11	Cs_2_CO_3_	2.25	1.50	81	10	8
12	Cs_2_CO_3_	2.50	1.50	72	12	15

*^a^***1a** (5.00 mmol), **2a** (5.50 mmol), [RuCl_2_(cod)]_n_ (0.25 mol.%), **L4** (1.25 mol.%), base (x mol.%), toluene (y mL), 120 °C, 2 h of catalyst generation time, and 36 h of reaction time; *^b^* NMR yields (average of two consistent runs) using 1,3,5-trimethoxybenzene as an internal standard.

## Supplementary Materials

Supplementary materials, which contain ^1^H-NMR and ^13^C-NMR data, as well as spectra of amides **3a**–**3t**, are available online.
