# Supplementary material for: Ruthenium-Based Catalytic Systems Incorporating a Labile Cyclooctadiene Ligand with N-Heterocyclic Carbene Precursors for the Atom-Economic Alcohol Amidation Using Amines
Source: Molecules. 2018 Sep 20;23(10):2413. doi: 10.3390/molecules23102413 (PMC6222456; doi:10.3390/molecules23102413)

Supporting Information for

Ruthenium-Based Catalytic Systems Incorporating a Labile Cyclooctadiene Ligand with N-Heterocyclic Carbene Precursors for the Atom-Economic Alcohol Amidation Using Amines

Cheng Chen,[1] Yang Miao,[2] Kimmy De Winter,[3] Hua-Jing Wang,[4] Patrick Demeyere,[3] Ye Yuan,*[1] and Francis Verpoort*[1, 2, 5, 6]

[1] State Key Laboratory of Advanced Technology for Materials Synthesis and Processing, Wuhan University of Technology, 122 Luoshi Road, Wuhan 430070, P. R. China;

[2] School of Materials Science and Engineering, Wuhan University of Technology, 122 Luoshi Road, Wuhan 430070, P. R. China;

[3] Odisee/KU Leuven Technology Campus, Gebroeders de Smetstraat 1, 9000 Ghent, Belgium;

[4] School of Chemistry, Chemical Engineering and Life Sciences, Wuhan University of Technology, 122 Luoshi Road, Wuhan 430070, P. R. China;

[5] National Research Tomsk Polytechnic University, Lenin Avenue 30, Tomsk 634050, Russian Federation

[6] Ghent University Global Campus, 119 Songdomunhwa-Ro, Yeonsu-Gu, Incheon 21985, Korea

Contents:

1. Characterization data for amides **3a-3t**.. S2-S6

2. Original ^{1}H and ^{13}C NMR spectra for amides **3a-3t**... S7-S26

1. Characterization data for amides 3a-3t

*N-benzylbenzamide (**3a**).*[1] Isolated yield: 86%. ^{1}H NMR (500 MHz, $CDCl_3$) δ 7.87 – 7.77 (m, 2H), 7.57 – 7.50 (m, 1H), 7.48 – 7.41 (m, 2H), 7.38 (d, *J* = 4.4 Hz, 4H), 7.36 – 7.29 (m, 1H), 6.45 (brs, 1H), 4.68 (d, *J* = 5.6 Hz, 2H). ^{13}C NMR (126 MHz, $CDCl_3$) δ 167.3, 138.2, 134.4, 131.6, 128.8, 128.6, 127.9, 127.6, 126.9, 44.2.

*N-hexylbenzamide (**3b**).*[1] Isolated yield: 85%. ^{1}H NMR (500 MHz, $CDCl_3$) δ 7.86 – 7.73 (m, 2H), 7.58 – 7.46 (m, 1H), 7.46 – 7.38 (m, 2H), 6.30 (brs, 1H), 3.65 – 3.29 (m, 2H), 1.76 – 1.55 (m, 2H), 1.48 – 1.25 (m, 6H), 1.02 – 0.76 (m, 3H). ^{13}C NMR (126 MHz, $CDCl_3$) δ 167.5, 134.9, 131.3, 128.5, 126.8, 40.1, 31.5, 29.6, 26.7, 22.6, 14.0.

*N-phenethylbenzamide (**3c**).* [1] Isolated yield: 95%. ^{1}H NMR (500 MHz, $CDCl_3$) δ 7.72 (dd, *J* = 7.4, 2.5 Hz, 2H), 7.54 – 7.47 (m, 1H), 7.45 – 7.39 (m, 2H), 7.38 – 7.32 (m, 2H), 7.29 – 7.24 (m, 3H), 6.32 (brs, 1H), 3.78 – 3.68 (m, 2H), 2.96 (t, *J* = 6.9 Hz, 2H). ^{13}C NMR (126 MHz, $CDCl_3$) δ 167.5, 138.9, 134.7, 131.4, 128.8, 128.7, 128.5, 126.8, 126.6, 41.2, 35.7.

*N-benzylhexanamide (**3d**).* [2] Isolated yield: 80%. ^{1}H NMR (500 MHz, $CDCl_3$) δ 7.40 – 7.33 (m, 2H), 7.33 – 7.29 (m, 3H), 5.71 (brs, 1H), 4.47 (d, *J* = 5.7 Hz, 2H), 2.23 (t, *J* = 7.6 Hz, 2H), 1.74 – 1.65 (m, 2H), 1.40 – 1.30 (m, 4H), 0.92 (t, *J* = 6.8 Hz, 3H). ^{13}C NMR (126 MHz, $CDCl_3$) δ 172.9, 138.4, 128.7, 127.8, 127.5, 43.6, 36.8, 31.5, 25.5, 22.4, 13.9.

*N-benzyl-2-phenylacetamide (**3e**).*[1] Isolated yield: 81%. ^{1}H NMR (500 MHz, $CDCl_3$) δ 7.41 – 7.35 (m, 2H), 7.35 – 7.25 (m, 6H), 7.23 – 7.18 (m, 2H), 5.76 (brs,

1H), 4.44 (d, *J* = 5.8 Hz, 2H), 3.65 (s, 2H). ^{13}C NMR (126 MHz, $CDCl_3$) δ 170.8, 138.1, 134.8, 129.5, 129.1, 128.7, 127.5, 127.43, 127.40, 43.9, 43.6.

N-benzyl-N-methylbenzamide ***(3f)***.[1] Isolated yield: 80%. ^{1}H NMR (500 MHz, $CDCl_3$) δ 7.52 – 7.30 (m, 9H), 7.20 (s, 1H), 4.88 – 4.41 (m, 2H), 3.20 – 2.78 (m, 3H). ^{13}C NMR (126 MHz, $CDCl_3$) δ 172.3, 171.6, 137.1, 136.6, 136.3, 129.6, 128.8, 128.4, 128.2, 127.6, 127.0, 126.8, 55.2, 50.8, 37.0, 33.2.

Piperidin-2-one ***(3g)***. [1] Isolated yield: 73%. ^{1}H NMR (500 MHz, $CDCl_3$) δ 6.67 (brs, 1H), 3.31 (td, *J* = 5.9, 2.3 Hz, 2H), 2.36 (t, *J* = 6.5 Hz, 2H), 1.94 – 1.62 (m, 4H). ^{13}C NMR (126 MHz, $CDCl_3$) δ 172.4, 42.2, 31.6, 22.2, 20.8.

N-(4-methylbenzyl)benzamide ***(3h)***.[1] Isolated yield: 85%. ^{1}H NMR (500 MHz, $CDCl_3$) δ 7.81 (d, *J* = 7.1 Hz, 2H), 7.55 – 7.48 (m, 1H), 7.47 – 7.40 (m, 2H), 7.27 (d, *J* = 7.9 Hz, 2H), 7.18 (d, *J* = 7.8 Hz, 2H), 6.46 (brs, 1H), 4.62 (d, *J* = 5.5 Hz, 2H), 2.37 (s, 3H). ^{13}C NMR (126 MHz, $CDCl_3$) δ 167.3, 137.4, 135.2, 134.5, 131.5, 129.5, 128.6, 128.0, 127.0, 43.9, 21.1.

N-(4-fluorobenzyl)benzamide ***(3i)***.[1] Isolated yield: 75%. ^{1}H NMR (500 MHz, $CDCl_3$) δ 7.81 (d, *J* = 8.3 Hz, 2H), 7.52 (dd, *J* = 7.6, 7.4 Hz, 1H), 7.44 (dd, *J* = 7.8, 7.6 Hz, 2H), 7.40 – 7.33 (m, 2H), 7.04 (dd, *J* = 8.6, 8.4 Hz, 2H), 6.60 (brs, 1H), 4.62 (d, *J* = 5.8 Hz, 2H). ^{13}C NMR (126 MHz, $CDCl_3$) δ 167.4, 162.2 (d, *J* = 245.8 Hz), 134.3, 134.1 (d, *J* = 3.2 Hz), 131.6, 129.6 (d, *J* = 8.1 Hz), 128.6, 127.0, 115.6 (d, *J* = 21.5 Hz), 43.4.

N-(3-fluorobenzyl)benzamide ***(3j)***.[1] Isolated yield: 83%. ^{1}H NMR (500 MHz, $CDCl_3$) δ 7.83 – 7.77 (m, 2H), 7.55 – 7.48 (m, 1H), 7.47 – 7.39 (m, 2H), 7.36 – 7.28

(m, 1H), 7.12 (d, J = 7.6 Hz, 1H), 7.05 (d, J = 9.6 Hz, 1H), 6.97 (ddd, J = 8.6, 8.4, 2.6 Hz, 1H), 6.54 (brs, 1H), 4.64 (d, J = 5.9 Hz, 2H). ^{13}C NMR (126 MHz, $CDCl_3$) δ 167.4, 163.0 (d, J = 246.5 Hz), 140.8 (d, J = 6.1 Hz), 134.2, 131.7, 130.3 (d, J = 8.2 Hz), 128.6, 126.9, 123.3 (d, J = 3.0 Hz), 114.6 (d, J = 21.9 Hz), 114.5 (d, J = 21.2 Hz), 43.5.

*N-(2-fluorobenzyl)benzamide (**3k**).*[1] Isolated yields: 85%. ^{1}H NMR (500 MHz, $CDCl_3$) δ 7.80 (dd, J = 7.2, 1.5 Hz, 2H), 7.56 – 7.48 (m, 1H), 7.46 – 7.40 (m, 3H), 7.35 – 7.27 (m, 1H), 7.15 (ddd, J = 7.6, 7.4, 1.2 Hz, 1H), 7.13 – 7.05 (m, 1H), 6.55 (brs, 1H), 4.72 (d, J = 5.9 Hz, 2H). ^{13}C NMR (126 MHz, $CDCl_3$) δ 167.4, 161.2 (d, J = 245.8 Hz), 134.3, 131.6, 130.5 (d, J = 4.2 Hz), 129.46 (d, J = 8.1 Hz), 128.6, 127.0, 125.2 (d, J = 14.6 Hz), 124.4 (d, J = 3.6 Hz), 115.4 (d, J = 21.2 Hz), 38.2 (d, J = 3.8 Hz).

*N-benzyl-4-methylbenzamide (**3l**).*[1] Isolated yield: 95%. ^{1}H NMR (500 MHz, $CDCl_3$) δ 7.69 (d, J = 8.2 Hz, 2H), 7.35 (d, J = 4.5 Hz, 4H), 7.32 – 7.27 (m, 1H), 7.22 (d, J = 7.9 Hz, 2H), 6.39 (brs, 1H), 4.64 (d, J = 5.8 Hz, 2H), 2.39 (s, 3H). ^{13}C NMR (126 MHz, $CDCl_3$) δ 167.2, 142.0, 138.3, 131.5, 129.2, 128.8, 127.9, 127.6, 126.9, 44.1, 21.4.

*N-benzyl-4-fluorobenzamide (**3m**).*[1] Isolated yield: 76%. ^{1}H NMR (500 MHz, $CDCl_3$) δ 7.97 – 7.71 (m, 2H), 7.52 – 7.31 (m, 5H), 7.23 – 7.01 (m, 2H), 6.63 (brs, 1H), 4.88 – 4.43 (m, 2H). ^{13}C NMR (126 MHz, $CDCl_3$) δ 166.4, 164.8 (d, J = 251.8 Hz), 138.1, 130.6 (d, J = 3.2 Hz), 129.3 (d, J = 8.5 Hz), 128.8, 127.9, 127.7, 115.6 (d, J = 22.3 Hz), 44.2.

*N-benzyl-3-fluorobenzamide (**3n**)*.[1] Isolated yield: 85%. ^{1}H NMR (500 MHz, $CDCl_3$) δ 7.61 – 7.51 (m, 2H), 7.47 – 7.30 (m, 6H), 7.25 – 7.18 (m, 1H), 6.46 (brs, 1H), 4.66 (d, *J* = 5.7 Hz, 2H). ^{13}C NMR (126 MHz, $CDCl_3$) δ 166.0 (d, *J* = 2.4 Hz), 162.8 (d, *J* = 247.9 Hz), 137.9, 136.7 (d, *J* = 6.9 Hz), 130.2 (d, *J* = 7.8 Hz), 128.8, 127.9, 127.7, 122.4 (d, *J* = 3.1 Hz), 118.5 (d, *J* = 21.3 Hz), 114.4 (d, *J* = 23.0 Hz), 44.3.

*N-benzyl-2-fluorobenzamide (**3o**)*.[1] Isolated yield: 45%. ^{1}H NMR (500 MHz, $CDCl_3$) δ 8.17 (ddd, *J* = 8.0, 7.9, 1.9 Hz, 1H), 7.54 – 7.47 (m, 1H), 7.42 – 7.36 (m, 4H), 7.35 – 7.29 (m, 2H), 7.14 (ddd, *J* = 12.2, 8.3, 1.1 Hz, 1H), 7.06 (brs, 1H), 4.72 (dd, *J* = 5.8, 1.6 Hz, 2H). ^{13}C NMR (126 MHz, $CDCl_3$) δ 163.2 (d, *J* = 3.2 Hz), 160.7 (d, *J* = 246.8 Hz), 138.0, 133.3 (d, *J* = 9.4 Hz), 132.2 (d, *J* = 2.3 Hz), 128.8, 127.7, 127.6, 124.83 (d, *J* = 3.2 Hz), 121.0 (d, *J* = 11.4 Hz), 116.00 (d, *J* = 24.9 Hz), 44.1.

N-phenylbenzamide (***3p***). [1] Isolated yields: 25%. ^{1}H NMR (500 MHz, $CDCl_3$) δ 7.81 (d, *J* = 7.0 Hz, 2H), 7.72 (brs, 1H), 7.58 (d, *J* = 7.3 Hz, 2H), 7.49 (dd, *J* = 7.4, 7.2 Hz, 1H), 7.43 (dd, *J* = 7.6, 7.4 Hz, 2H), 7.36 – 7.26 (m, 2H), 7.09 (dd, *J* = 7.6, 7.4 Hz, 1H). ^{13}C NMR (126 MHz, $CDCl_3$) δ 165.7, 137.9, 135.0, 131.9, 129.1, 128.8, 127.0, 124.6, 120.2.

*N-benzylisobutyramide (**3q**)*. [2] Isolated yields: 78%. ^{1}H NMR (500 MHz, $CDCl_3$) δ 7.39 – 7.33 (m, 2H), 7.32 – 7.29 (m, 2H), 7.28 (s, 1H), 5.77 (brs, 1H), 4.46 (d, *J* = 5.7 Hz, 2H), 2.53 – 2.33 (m, 1H), 1.21 (d, *J* = 6.9 Hz, 6H). ^{13}C NMR (126 MHz, $CDCl_3$) δ 176.7, 138.5, 128.7, 127.7, 127.5, 43.5, 35.7, 19.6.

*N-benzyl-2-phenylpropanamide (**3r**)*.[1] Isolated yield: 81%. ^{1}H NMR (500 MHz,

$CDCl_3$) δ 7.58 – 7.21 (m, 8H), 7.17 (d, *J* = 6.9 Hz, 2H), 5.67 (brs, 1H), 4.53 – 4.29 (m, 2H), 3.62 (q, *J* = 7.2 Hz, 1H), 1.59 (d, *J* = 7.2 Hz, 3H). ^{13}C NMR (126 MHz, $CDCl_3$) δ 174.0, 141.3, 138.3, 129.0, 128.6, 127.7, 127.5, 127.4, 127.3, 47.2, 43.6, 18.5.

*N-(heptan-2-yl)benzamide (**3s**).* [2] Isolated yield: 85%. ^{1}H NMR (500 MHz, $CDCl_3$) δ 7.77 (dd, *J* = 6.9, 1.6 Hz, 2H), 7.53 – 7.46 (m, 1H), 7.42 (ddd, *J* = 8.1, 7.2, 2.2 Hz, 2H), 6.06 (brs, 1H), 4.19 (dq, *J* = 8.4, 6.6 Hz, 1H), 1.63 – 1.46 (m, 2H), 1.44 – 1.27 (m, 6H), 1.24 (d, *J* = 6.5 Hz, 3H), 0.90 (t, *J* = 6.4 Hz, 3H). ^{13}C NMR (126 MHz, $CDCl_3$) δ 166.7, 135.1, 131.1, 128.4, 126.8, 45.7, 37.0, 31.7, 25.7, 22.5, 21.0, 14.0.

*N-(1-phenylethyl)benzamide (**3t**).* White solid: m.p. 122.0-123.1 ^{o}C. Isolated yield: 75%. ^{1}H NMR (500 MHz, $CDCl_3$) δ 7.80 (d, *J* = 7.6 Hz, 2H), 7.57 – 7.33 (m, 7H), 7.32 (d, *J* = 7.2 Hz, 1H), 6.33 (brs, 1H), 5.45 – 5.27 (m, 1H), 1.64 (d, *J* = 6.9 Hz, 3H). ^{13}C NMR (126 MHz, $CDCl_3$) δ 166.5, 143.1, 134.6, 131.5, 128.8, 128.6, 127.5, 126.9, 126.3, 49.2, 21.7. HR-MS (ESI): m/z calcd. for $C_{15}H_{16}NO$ $[M+H]^+$: 226.1226; Found: 226.1228.

References

[1] H. Cheng, M. Q. Xiong, C. X. Cheng, H. J. Wang, Q. Lu, H. F. Liu, F. B. Yao, C. Chen, F. Verpoort, *Chem. Asian J.* **2018**, *13*, 440-448.

[2] H. Cheng, M. Q. Xiong, N. Zhang, H. J. Wang, Y. Miao, W. Su, Y. Yuan, C. Chen, F. Verpoort, *ChemCatChem*, DOI: 10.1002/cctc.201800945.

2. Original ^{1}H and ^{13}C NMR spectra for amides 3a-3t:

➢ ^{1}H NMR spectrum for **3a**

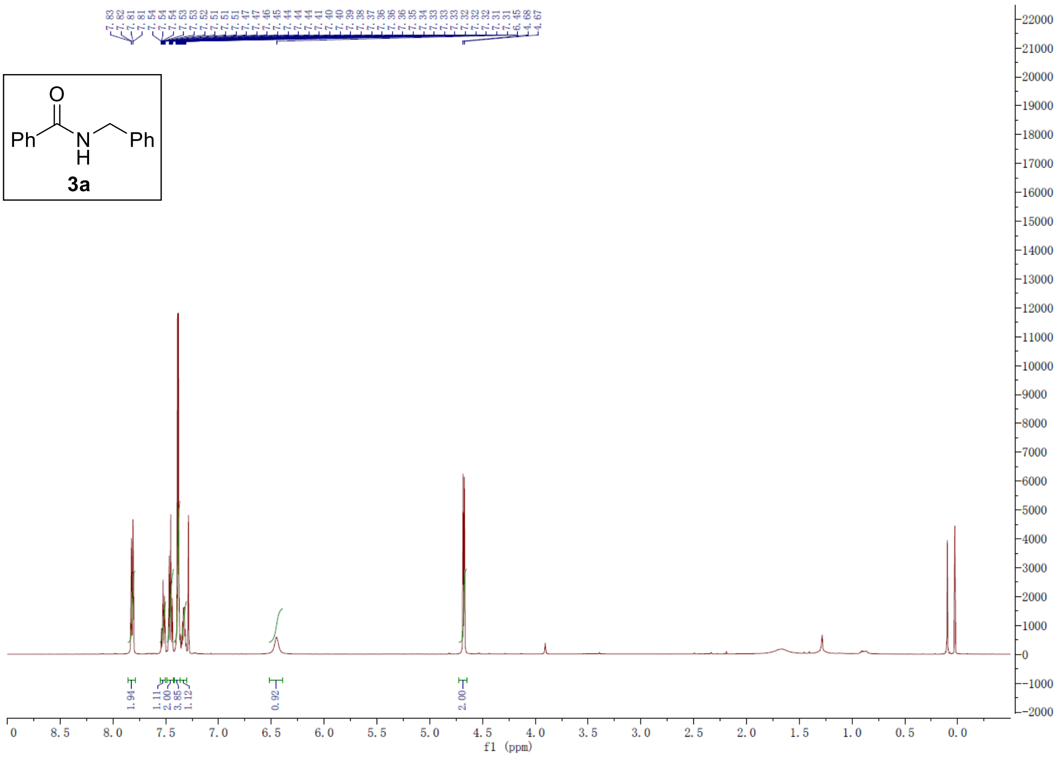

➢ ^{13}C NMR spectrum for **3a**

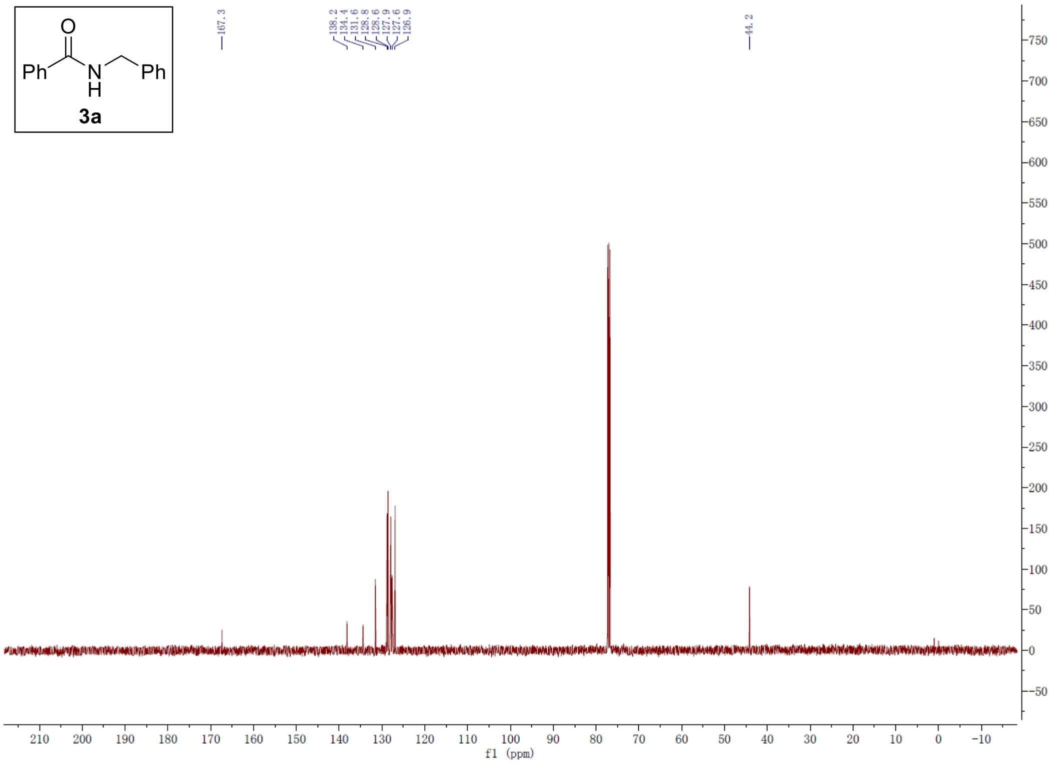

➢ ^{1}H NMR spectrum for **3b**

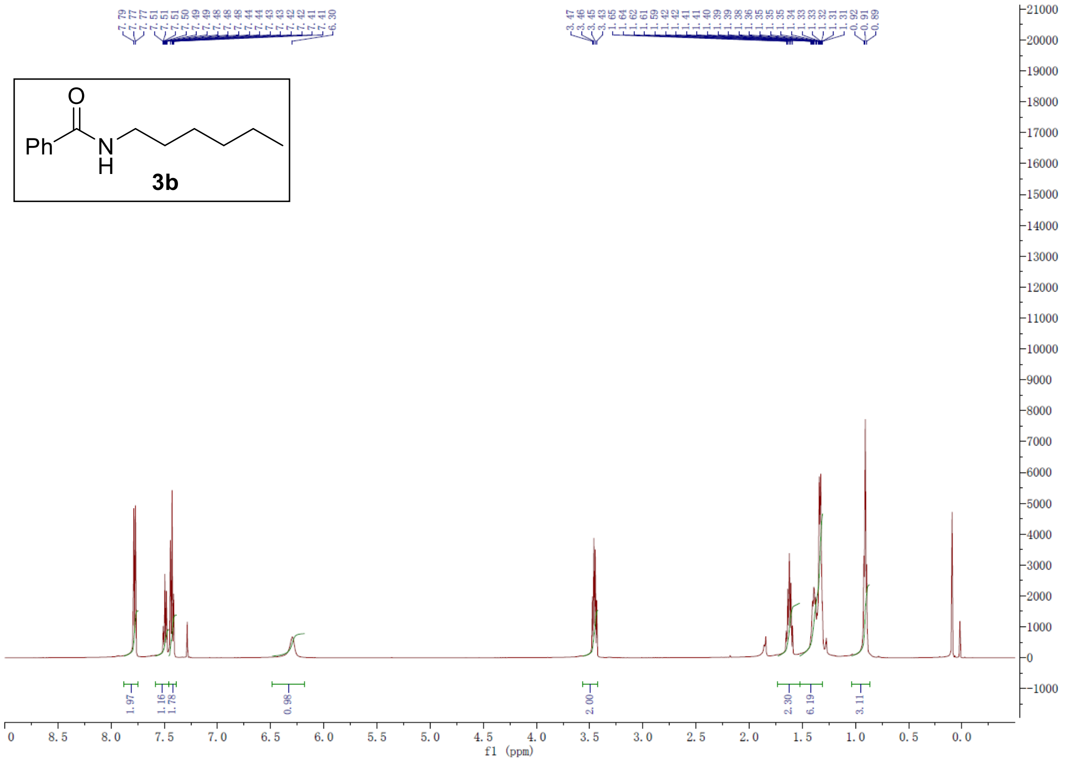

➢ ^{13}C NMR spectrum for **3b**

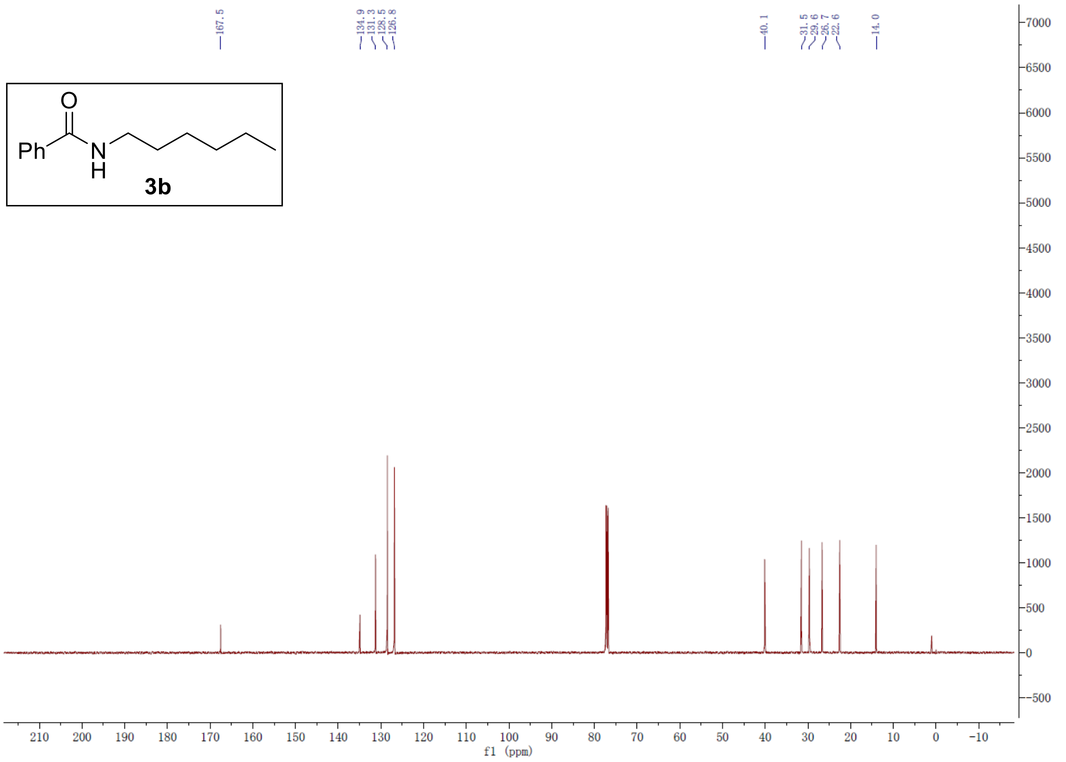

➢ ^{1}H NMR spectrum for **3c**

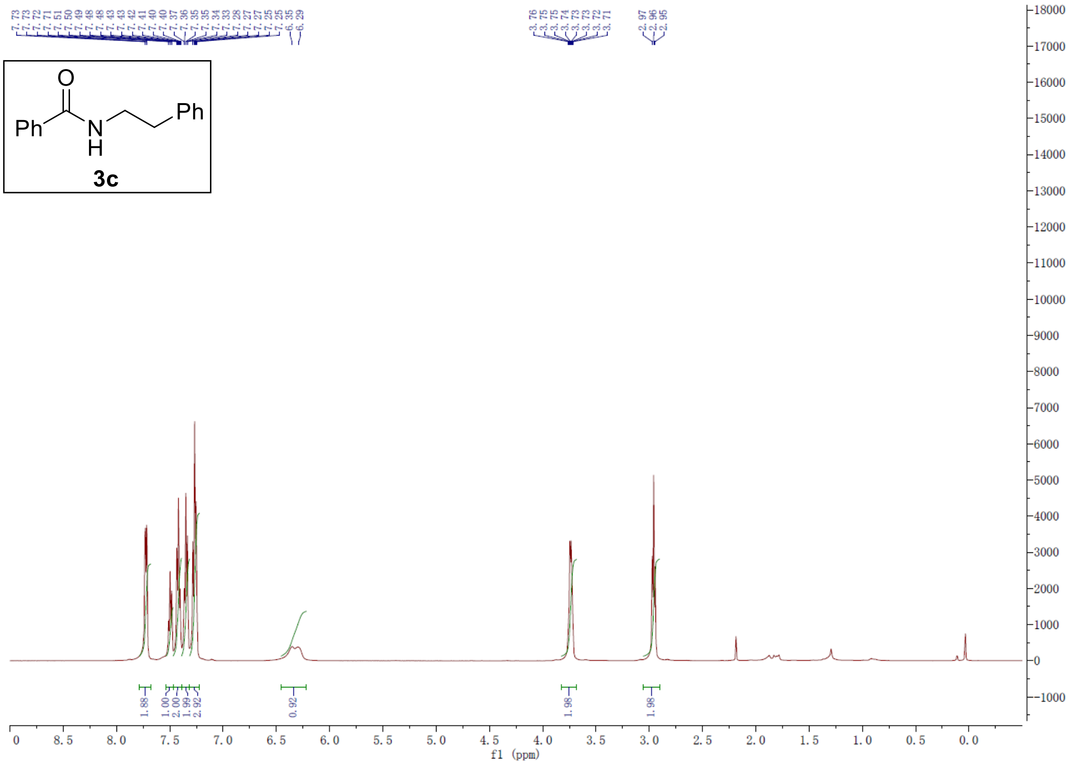

➢ ^{13}C NMR spectrum for **3c**

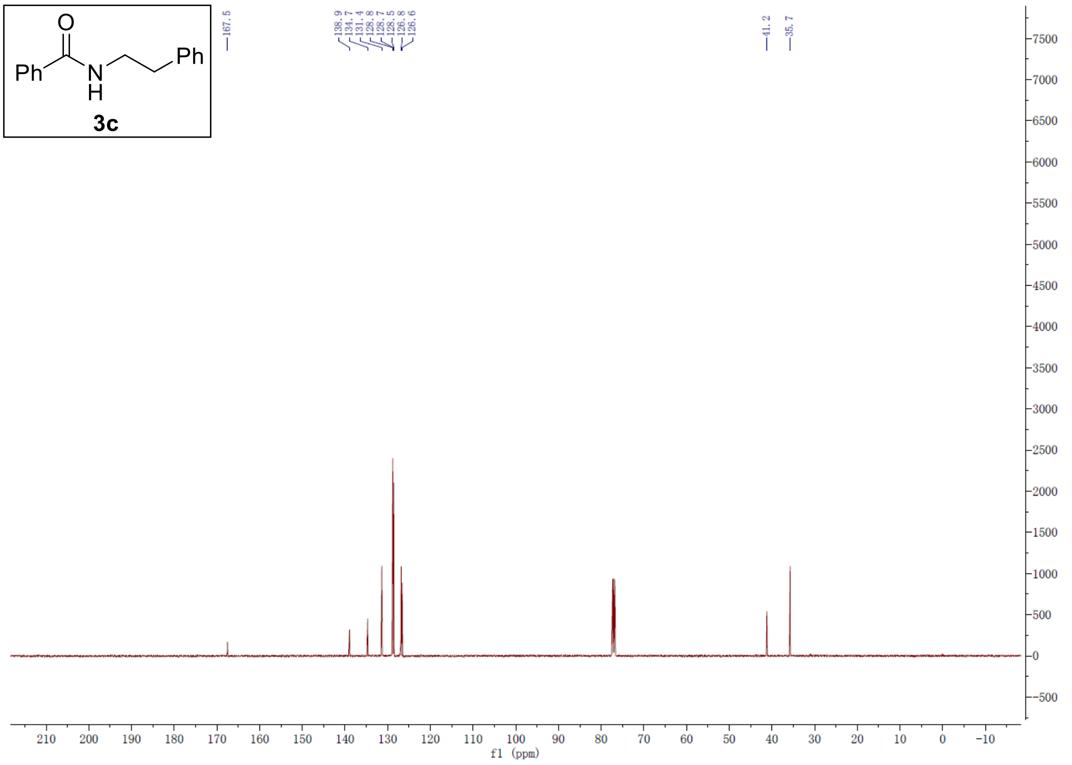

➢ ^{1}H NMR spectrum for **3d**

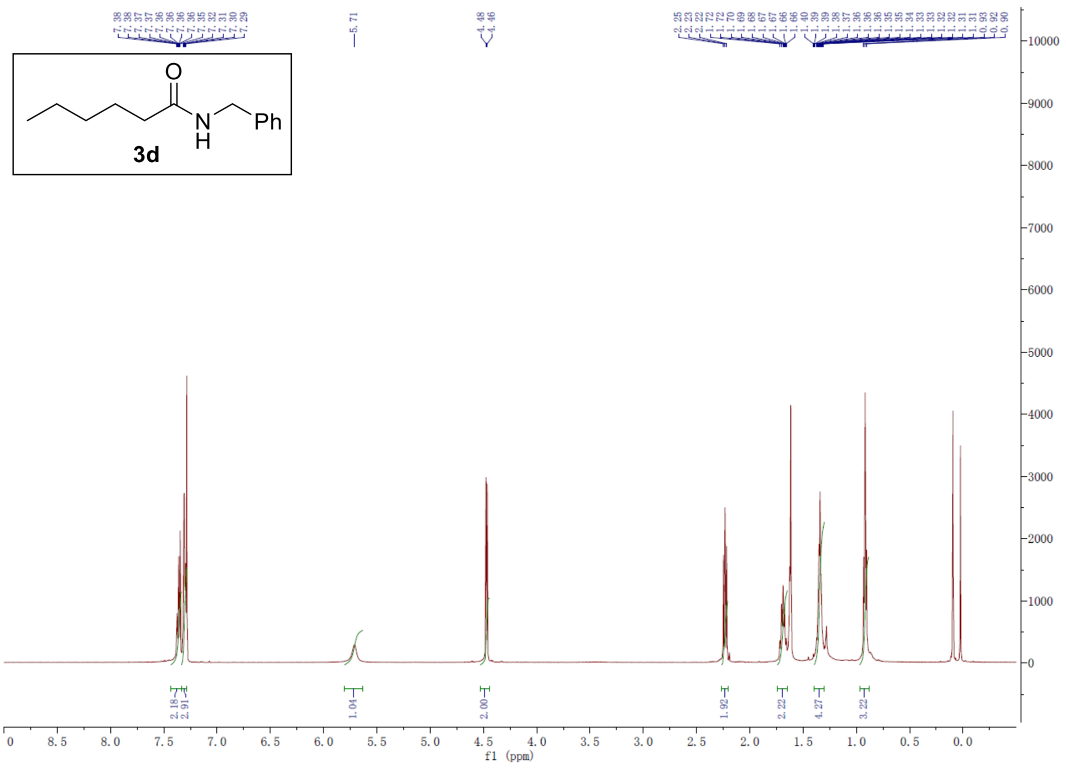

➢ ^{13}C NMR spectrum for **3d**

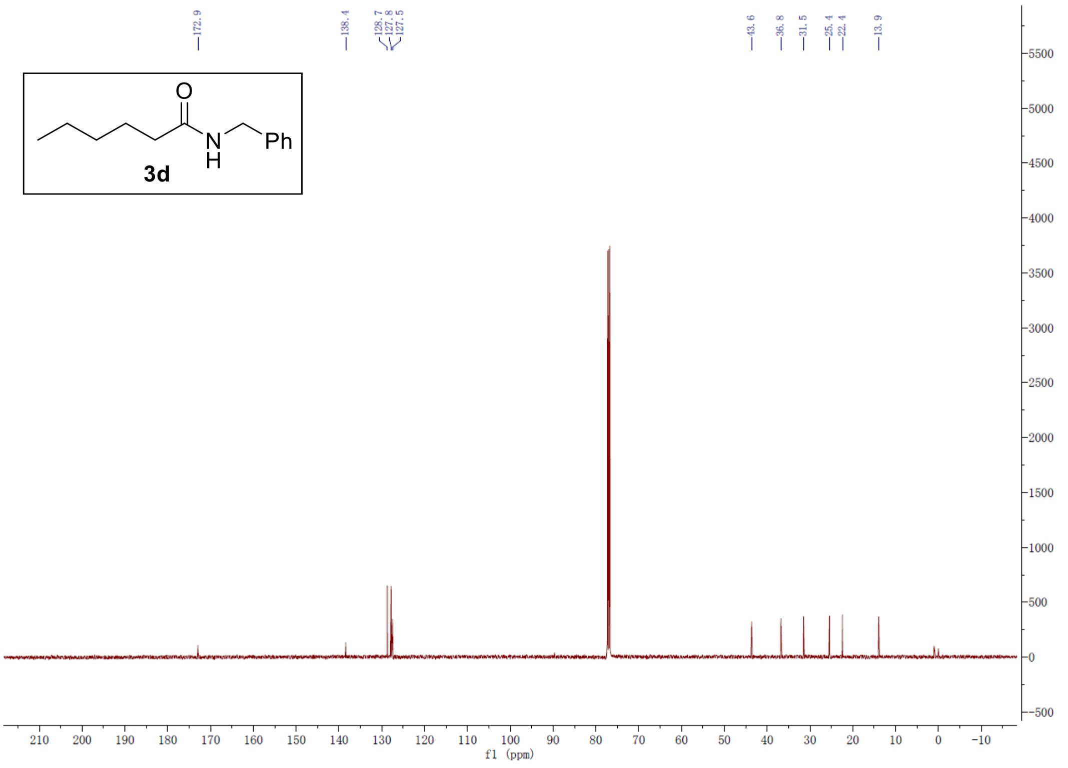

➢ ^{1}H NMR spectrum for **3e**

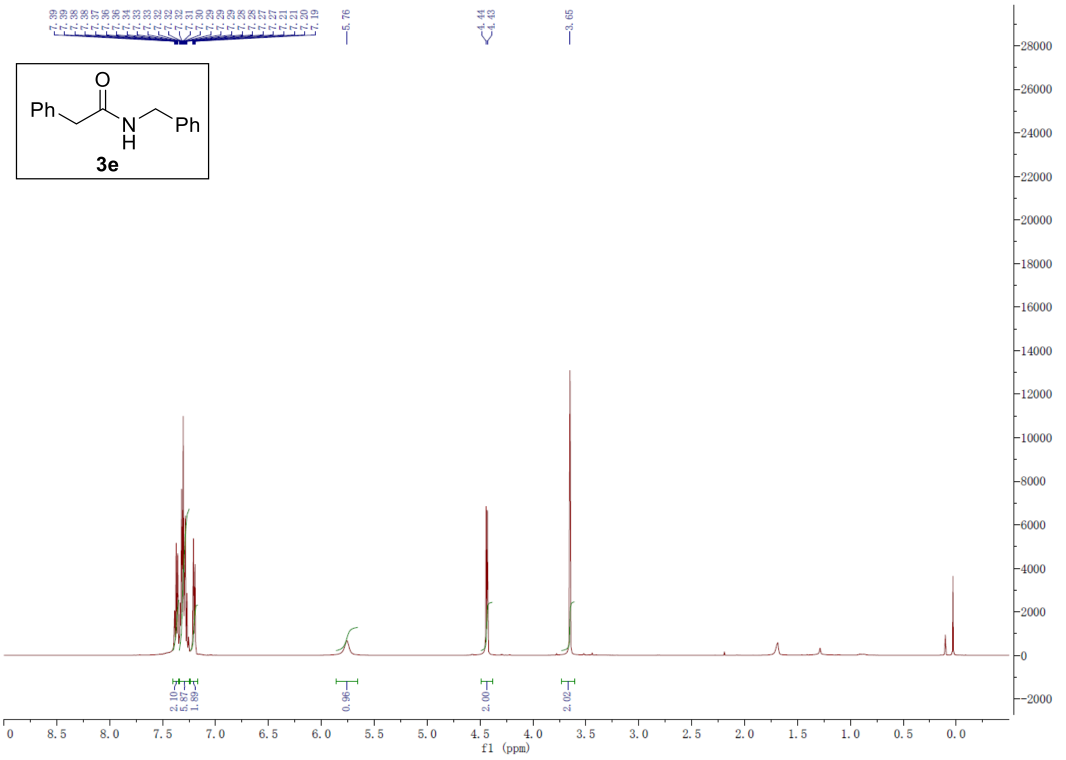

➢ ^{13}C NMR spectrum for **3e**

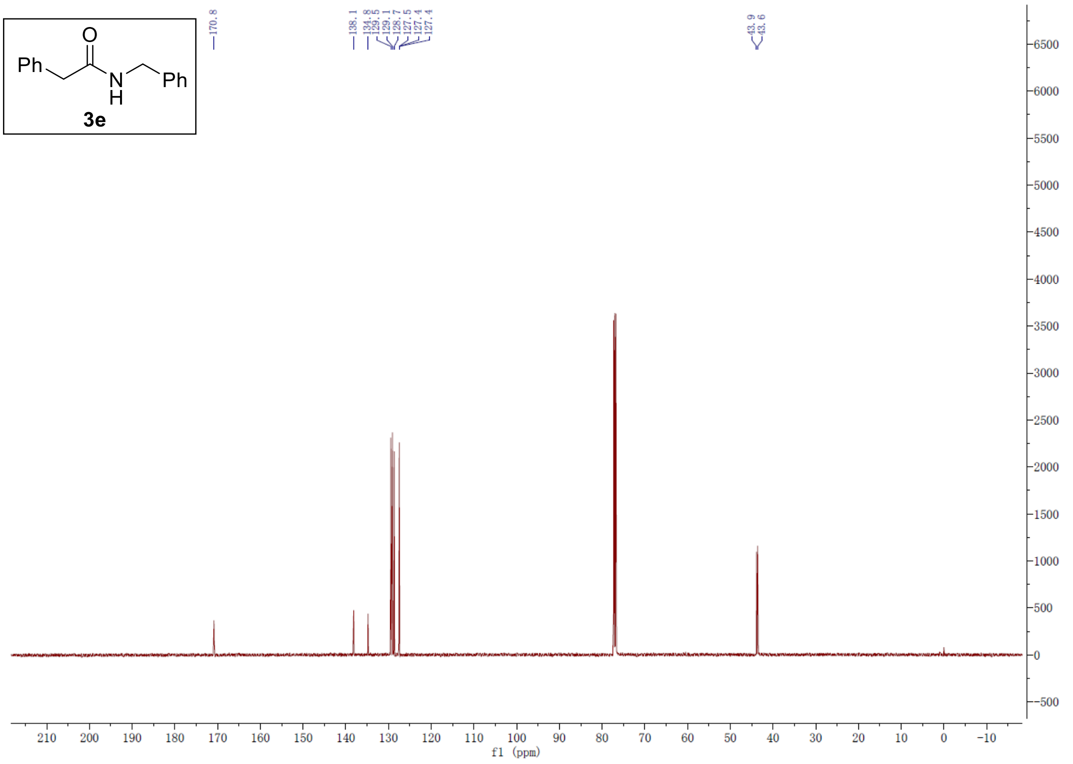

➢ ^{1}H NMR spectrum for **3f**

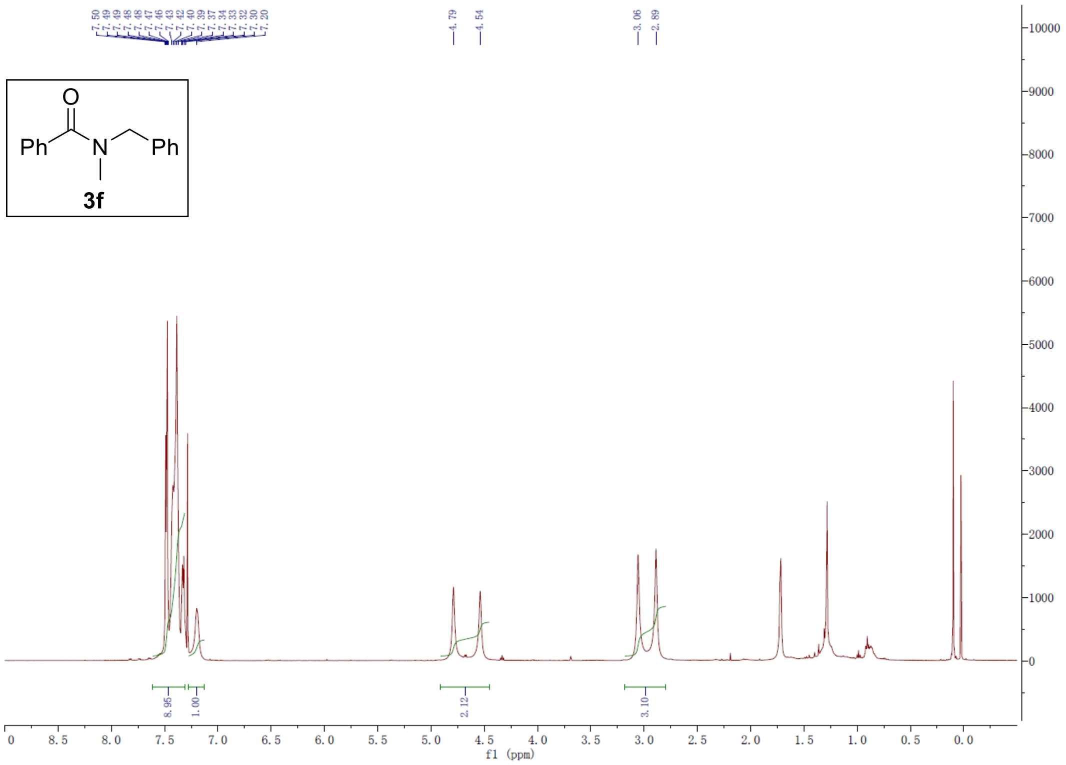

➢ ^{13}C NMR spectrum for **3f**

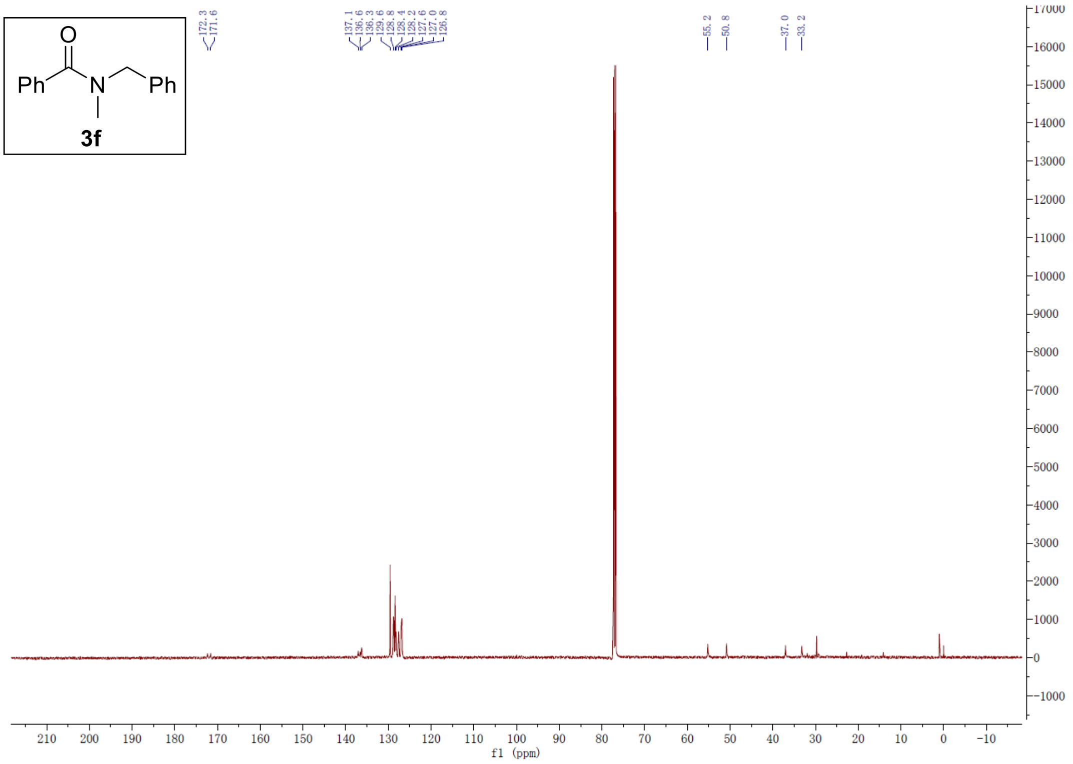

➢ ^{1}H NMR spectrum for **3g**

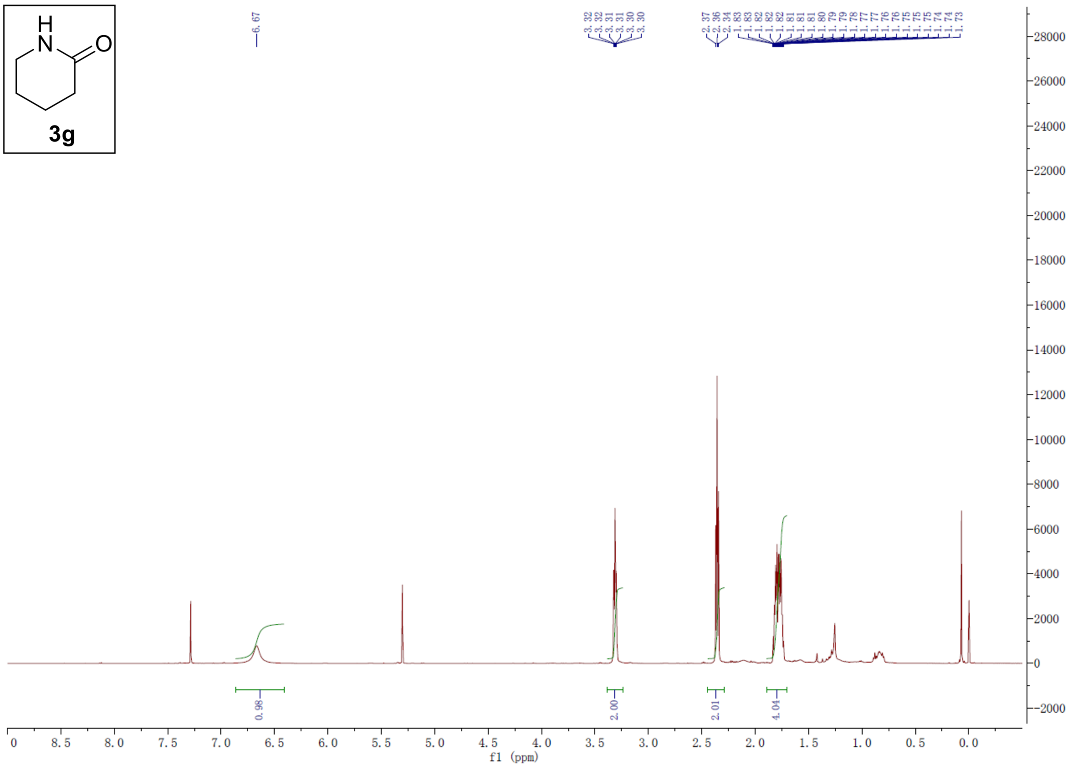

➢ ^{13}C NMR spectrum for **3g**

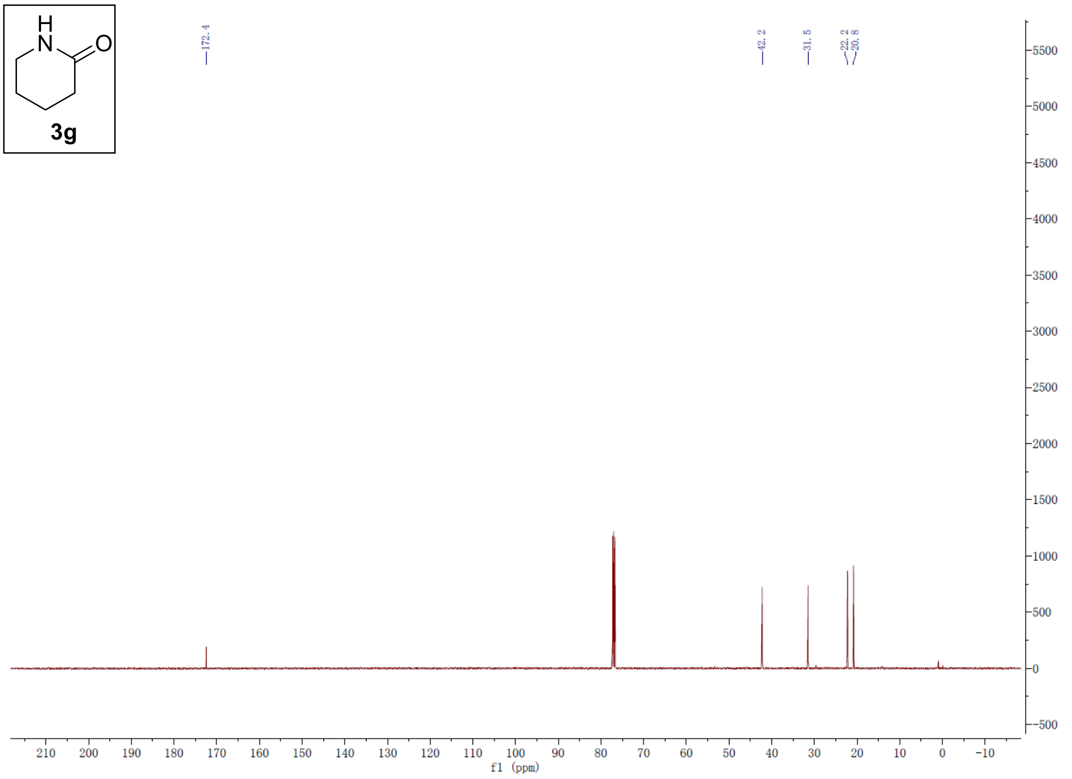

➢ ^{1}H NMR spectrum for **3h**

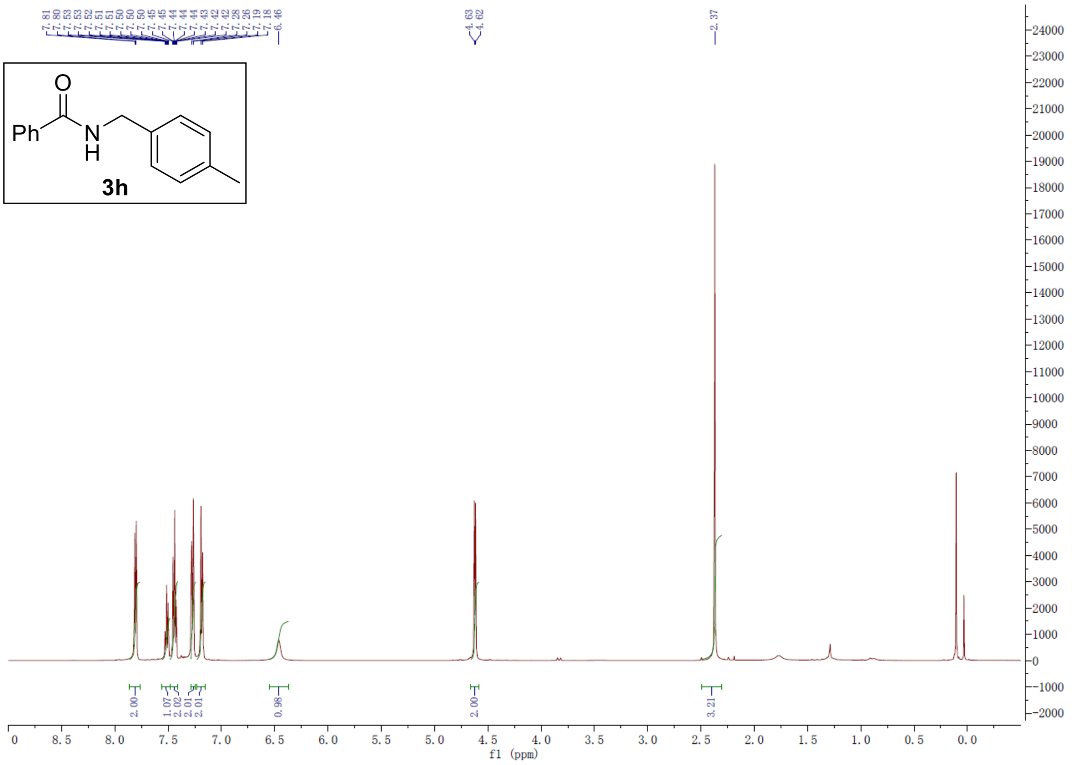

➢ ^{13}C NMR spectrum for **3h**

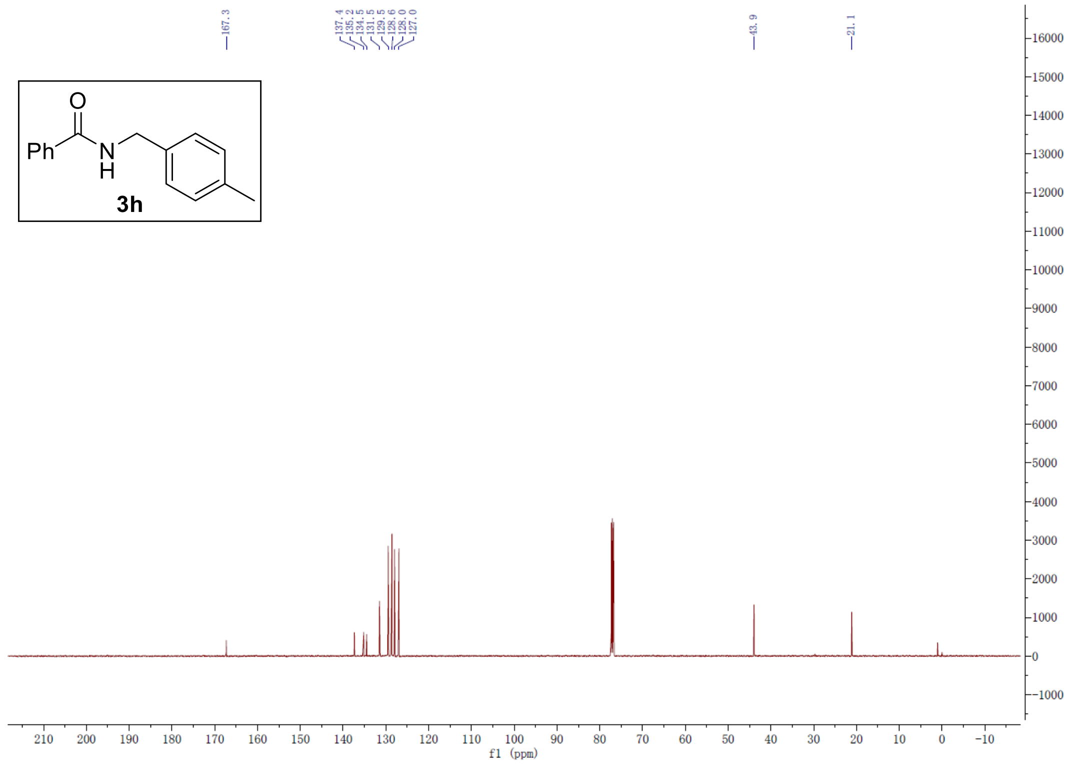

➢ ^{1}H NMR spectrum for **3i**

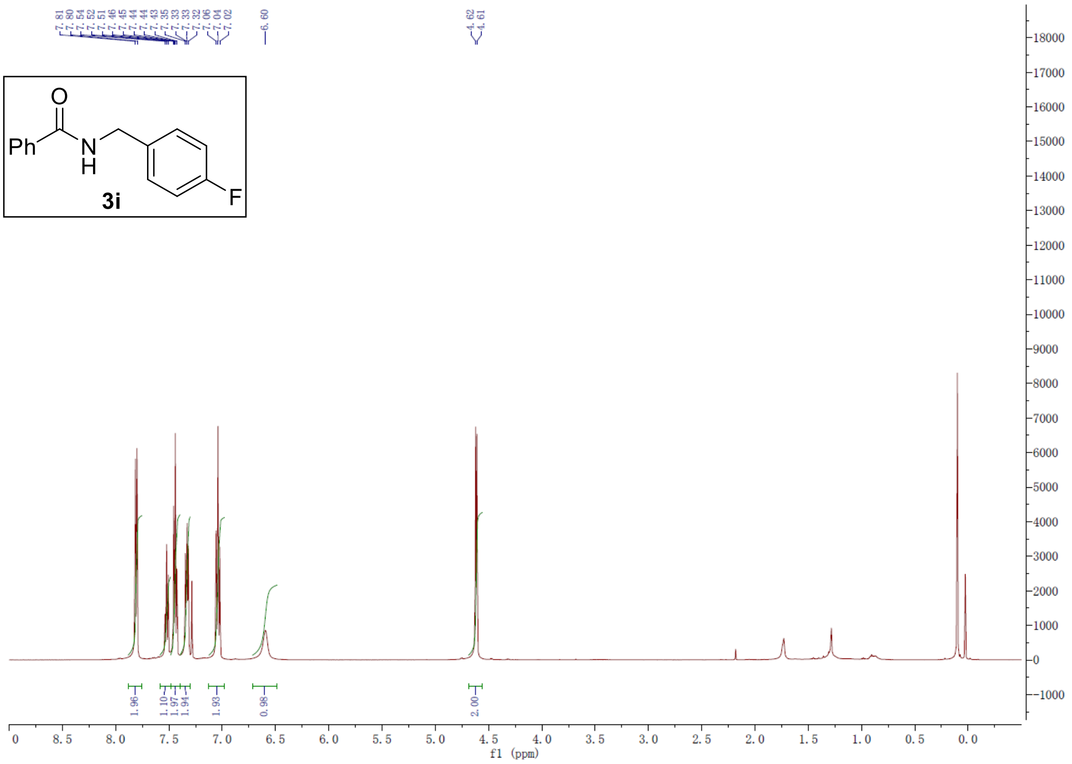

➢ ^{13}C NMR spectrum for **3i**

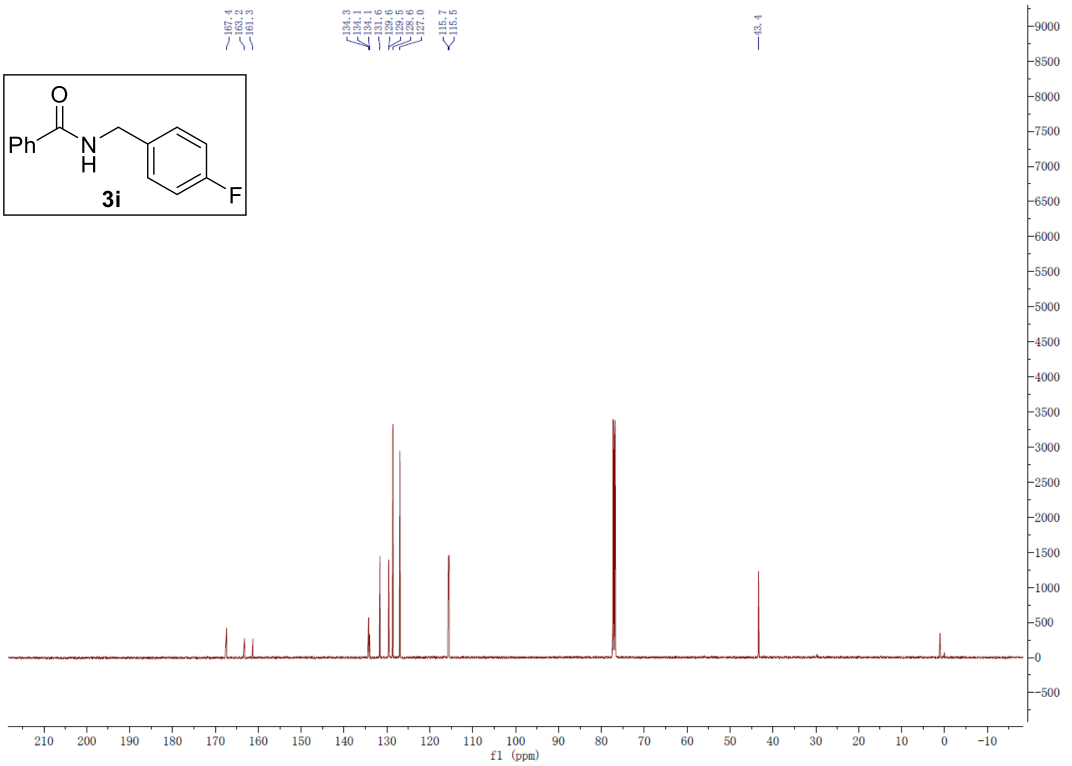

➢ ^{1}H NMR spectrum for **3j**

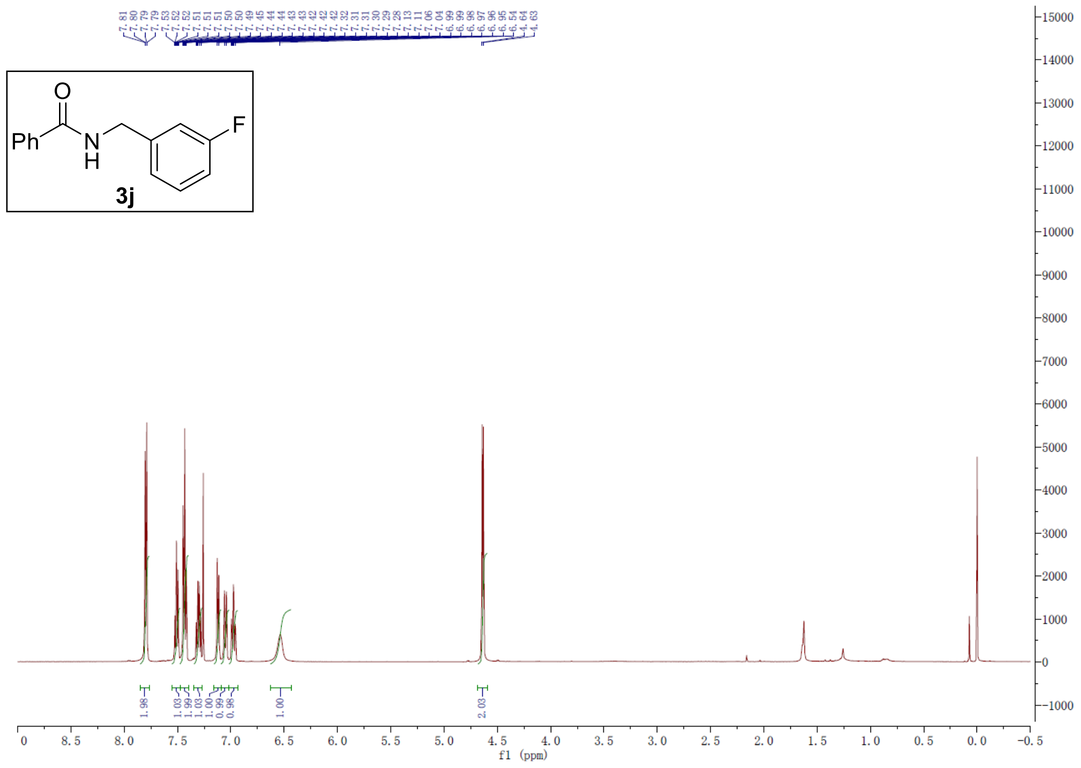

➢ ^{13}C NMR spectrum for **3j**

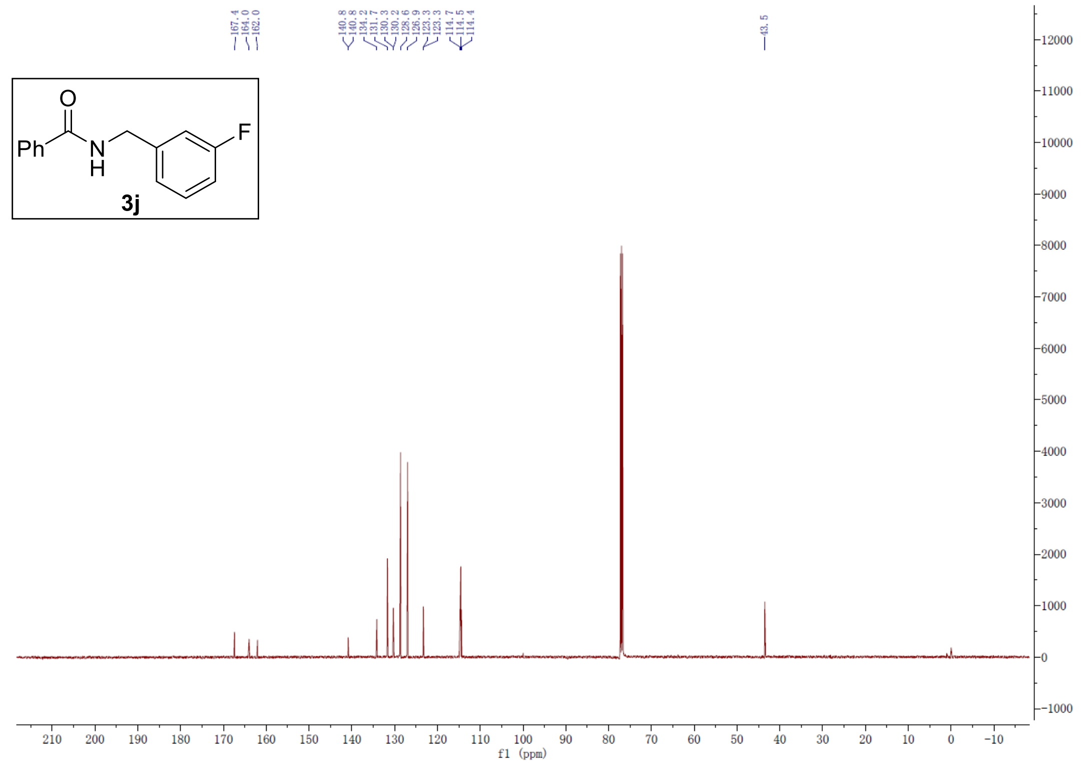

➢ ^{1}H NMR spectrum for **3k**

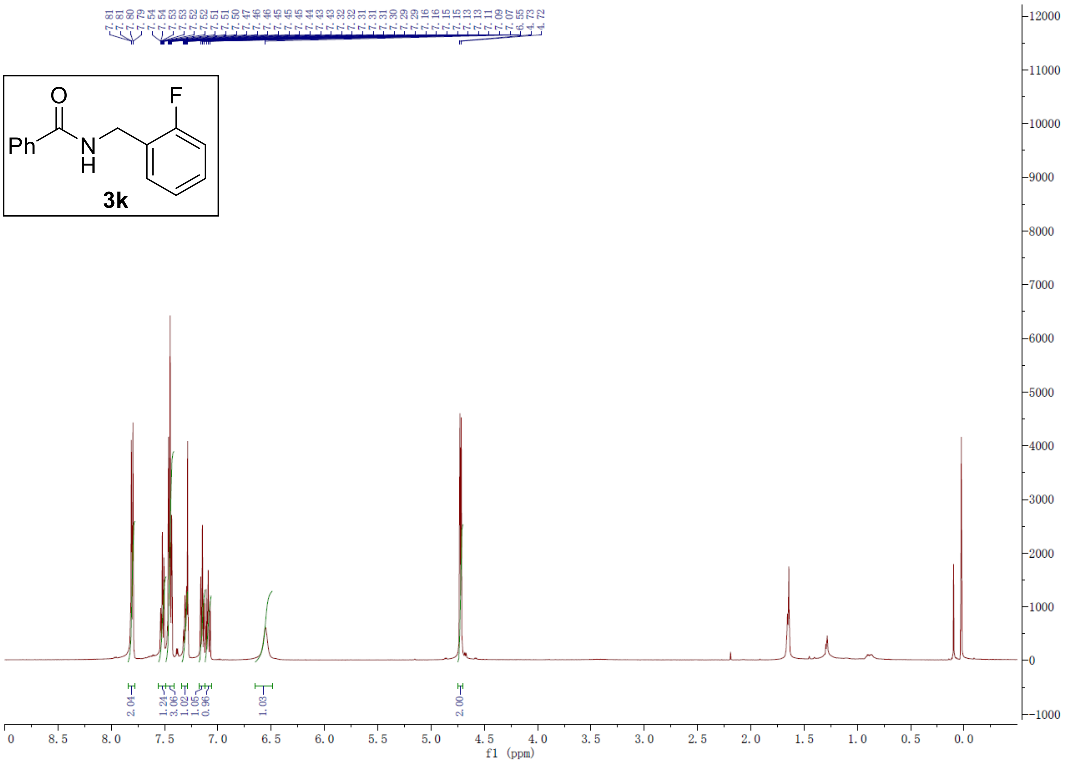

➢ ^{13}C NMR spectrum for **3k**

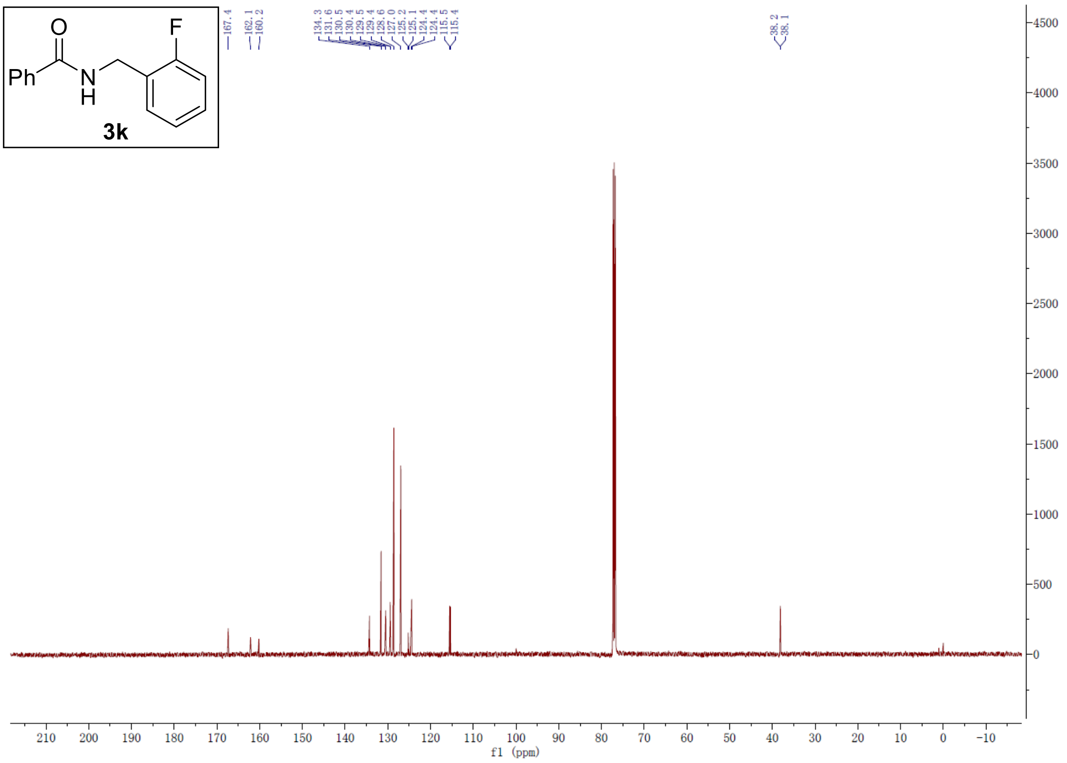

➢ ^{1}H NMR spectrum for **3l**

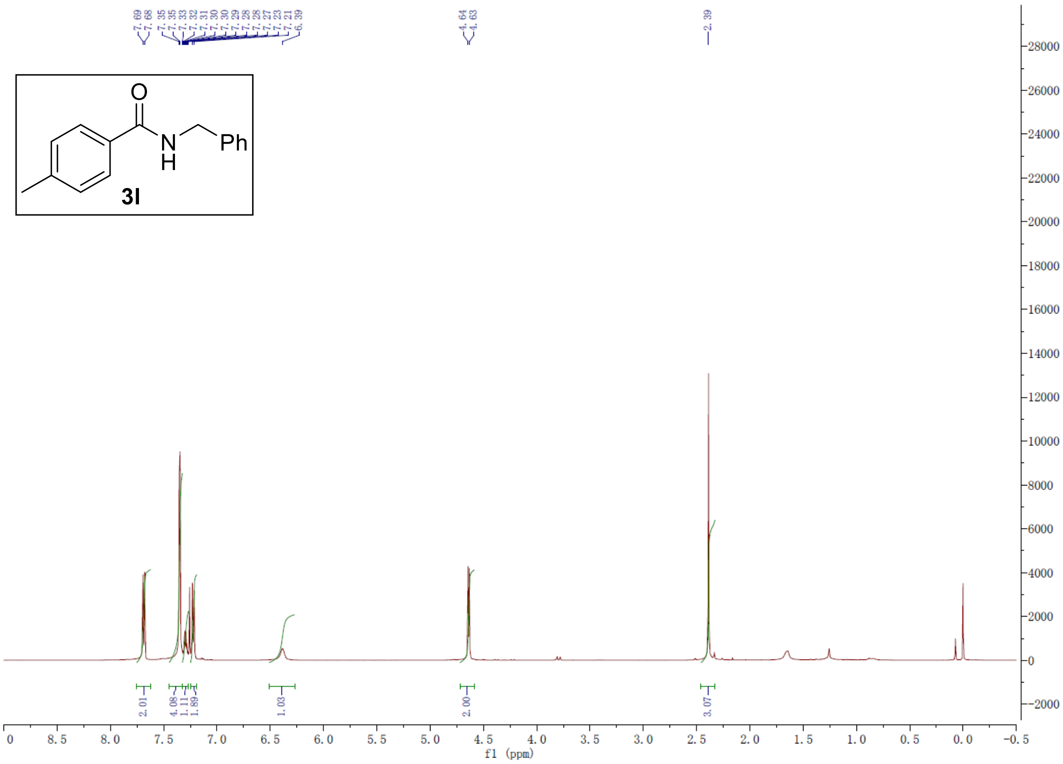

➢ ^{13}C NMR spectrum for **3l**

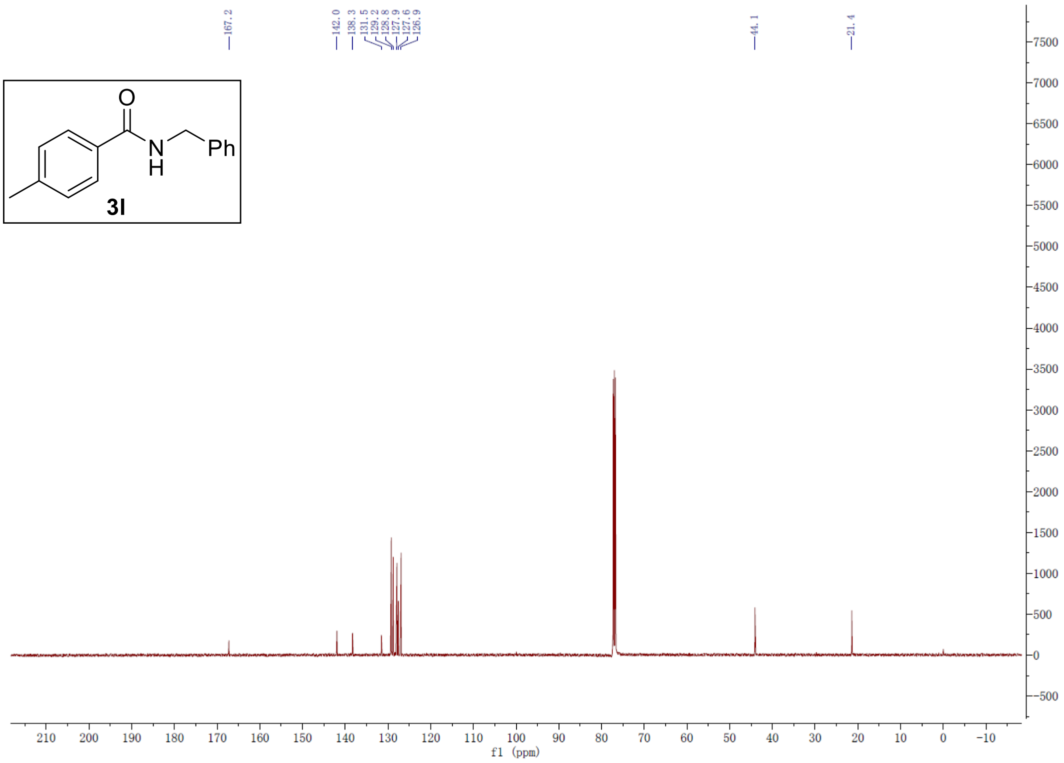

➢ ^{1}H NMR spectrum for **3m**

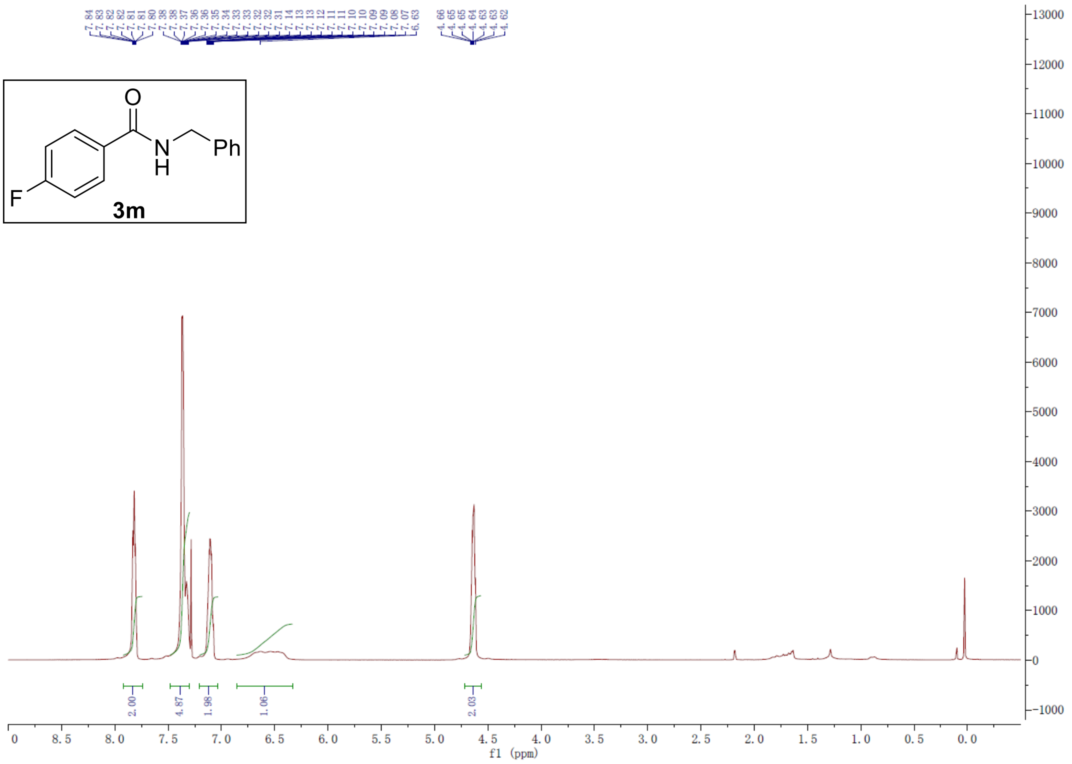

➢ ^{13}C NMR spectrum for **3m**

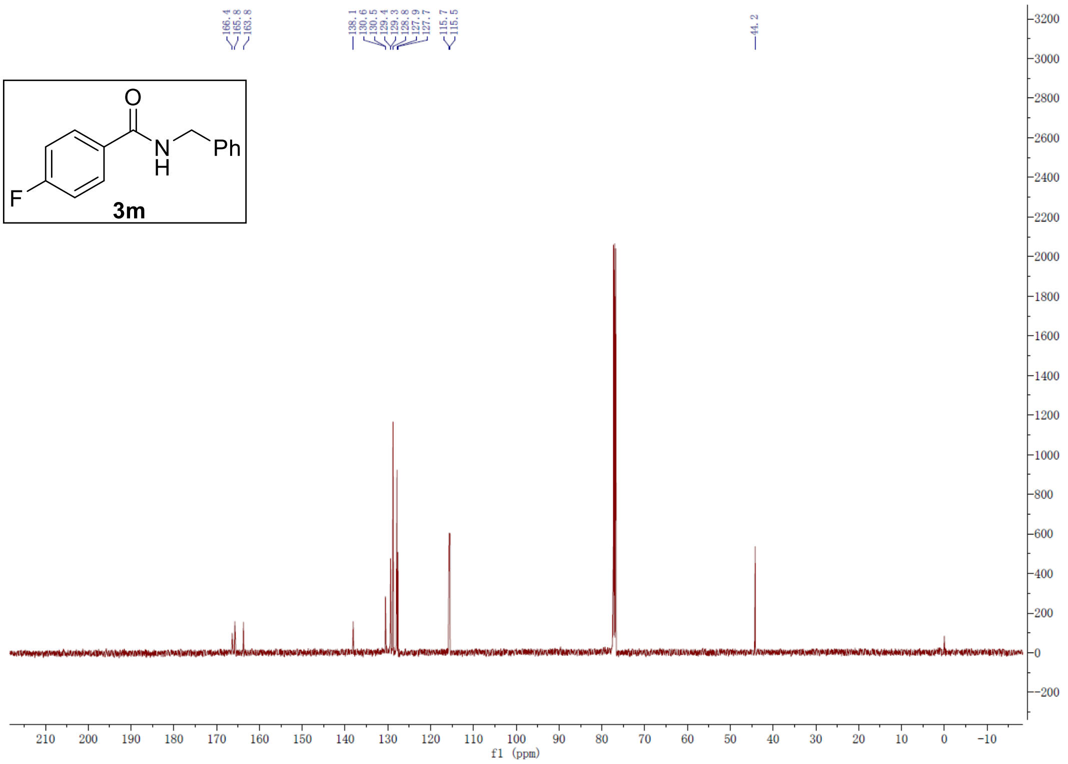

➢ ^{1}H NMR spectrum for **3n**

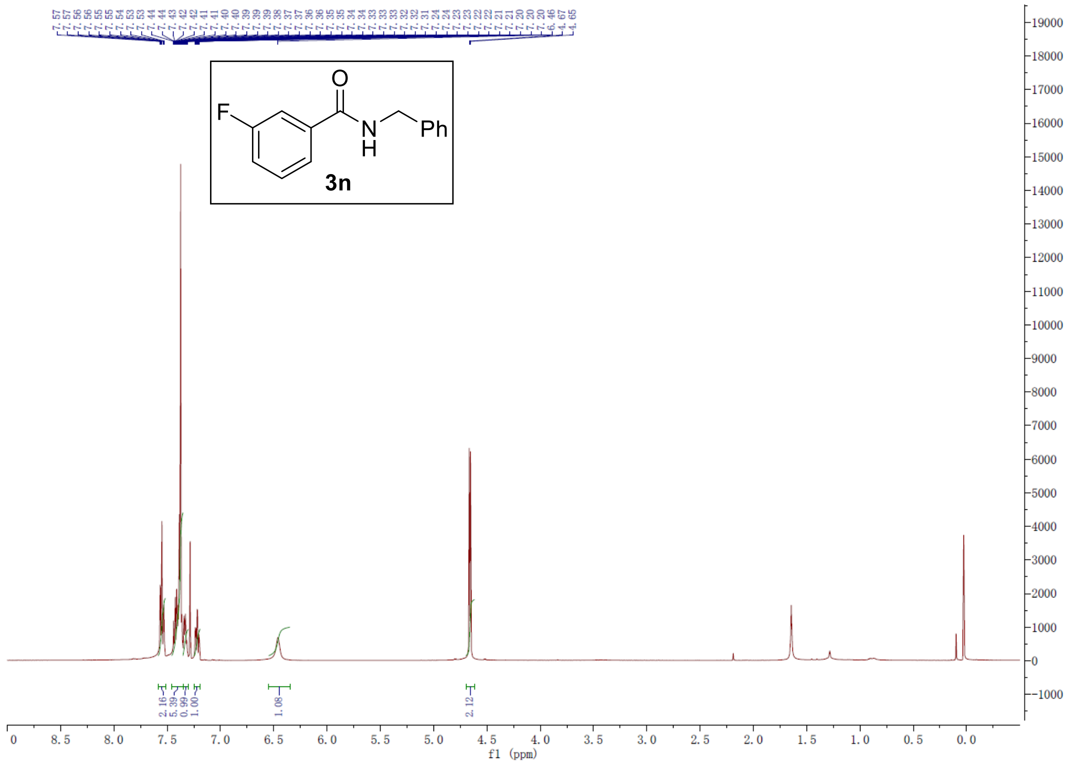

➢ ^{13}C NMR spectrum for **3n**

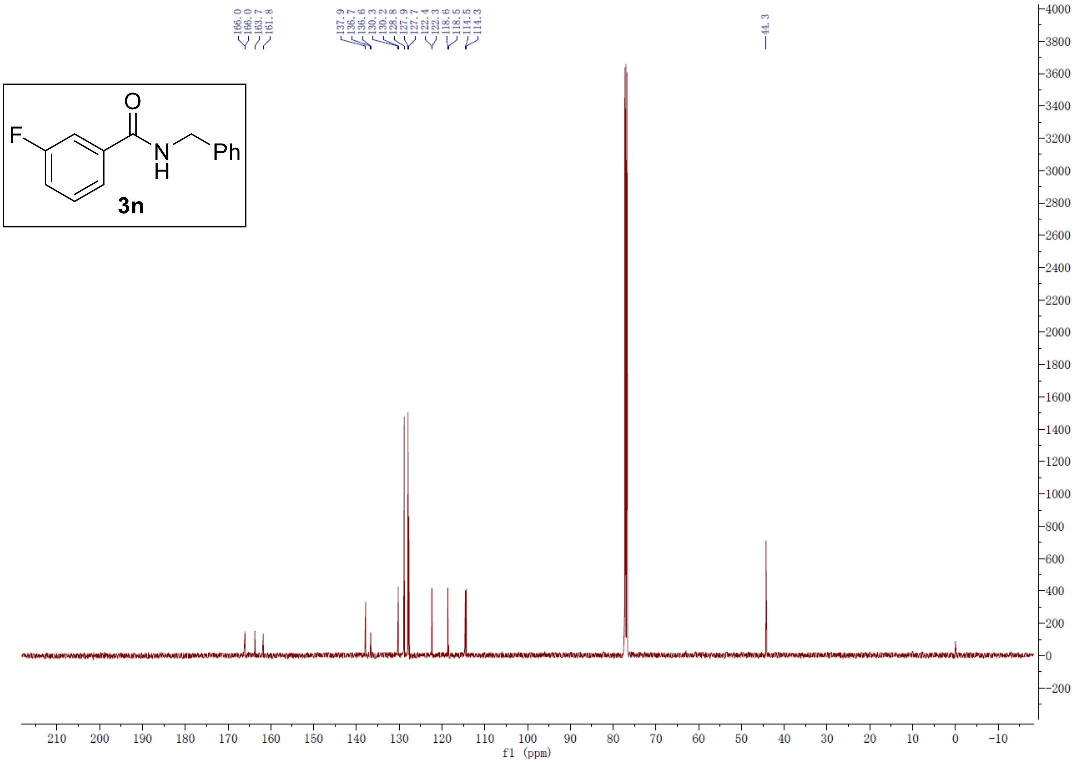

➢ ^{1}H NMR spectrum for **3o**

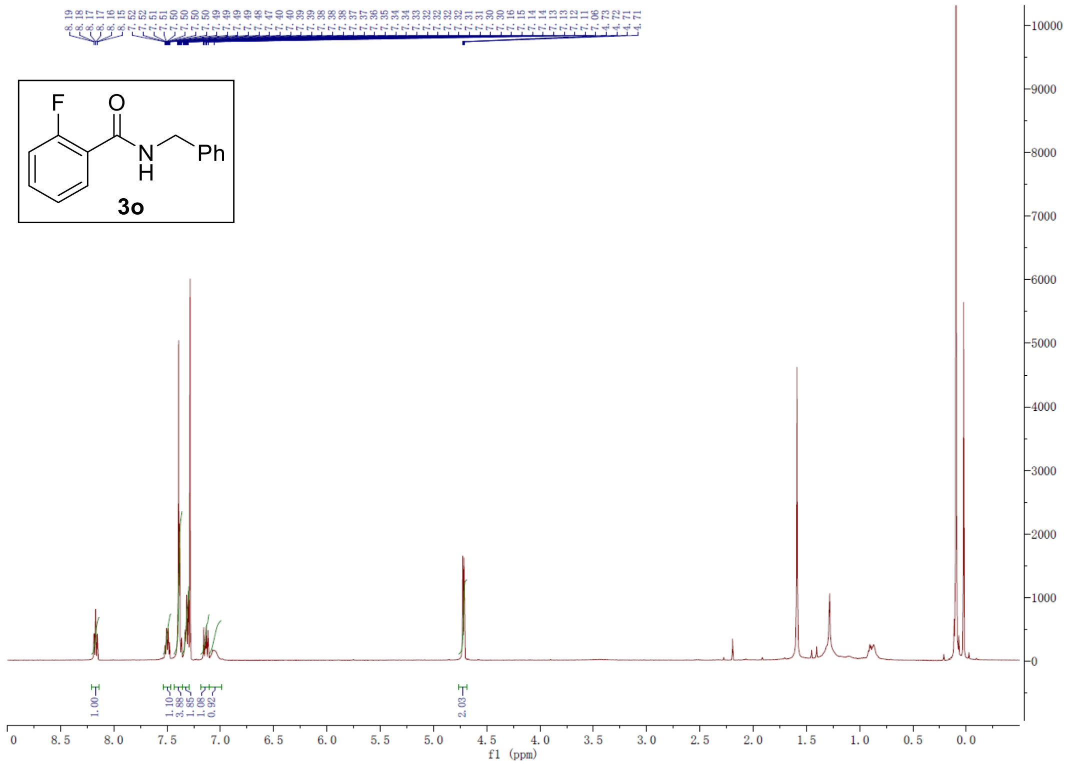

➢ ^{13}C NMR spectrum for **3o**

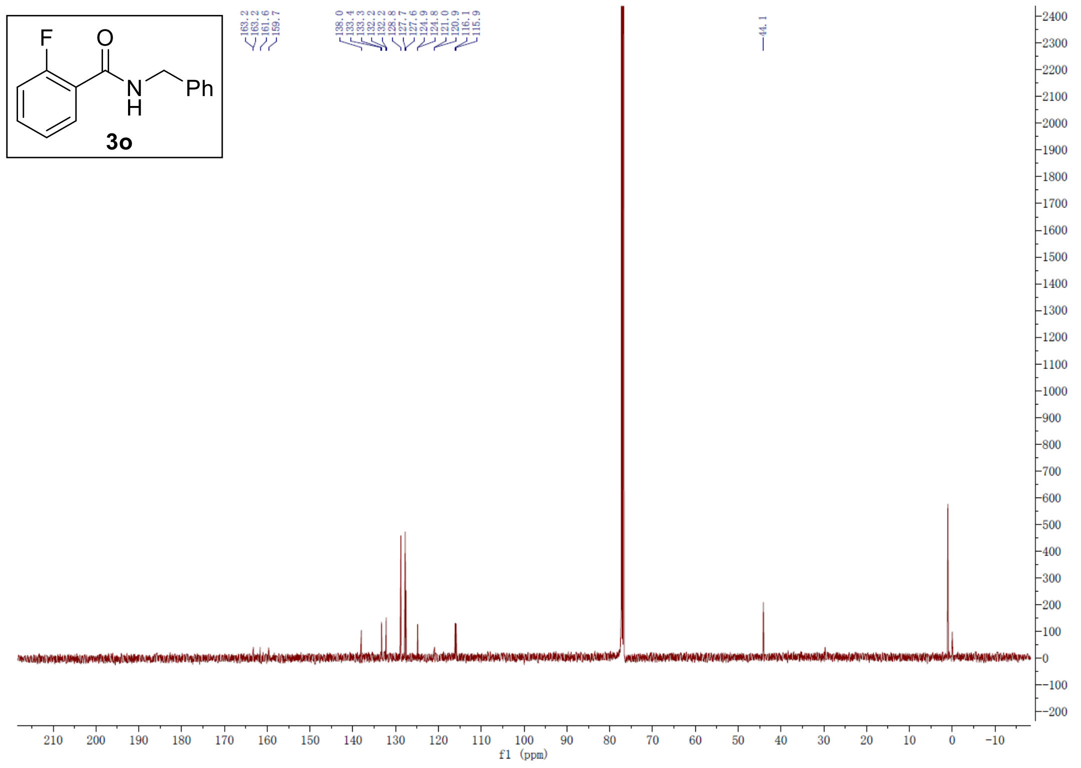

➢ ^{1}H NMR spectrum for **3p**

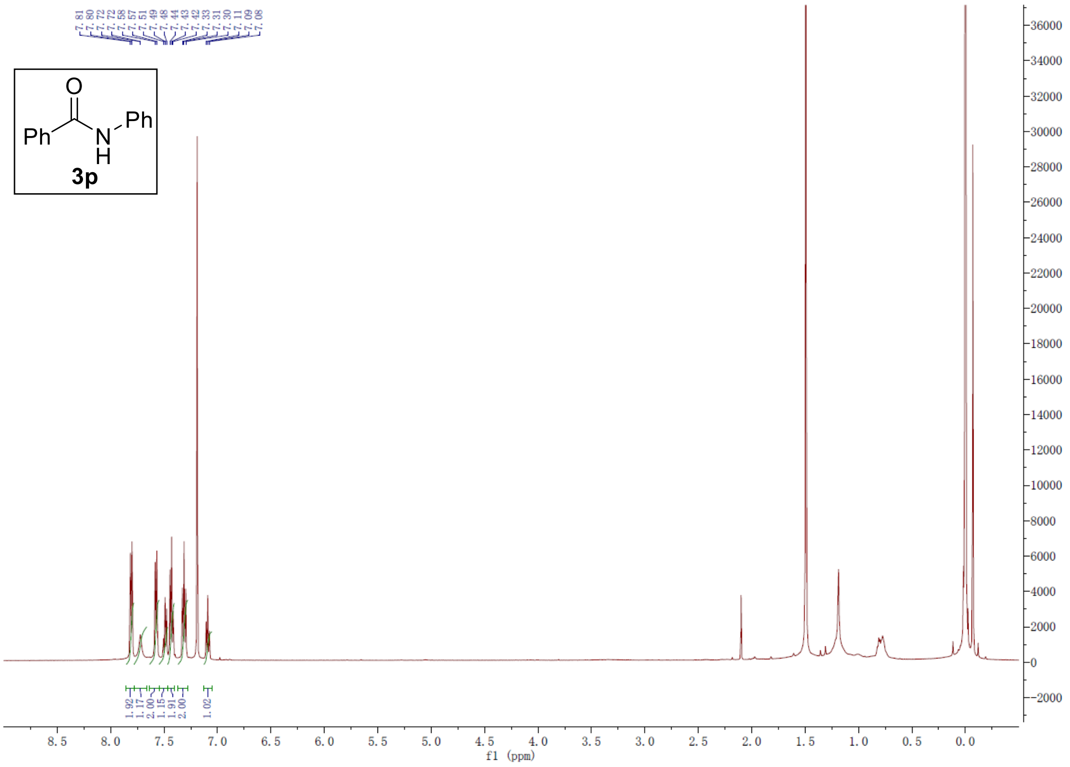

➢ ^{13}C NMR spectrum for **3p**

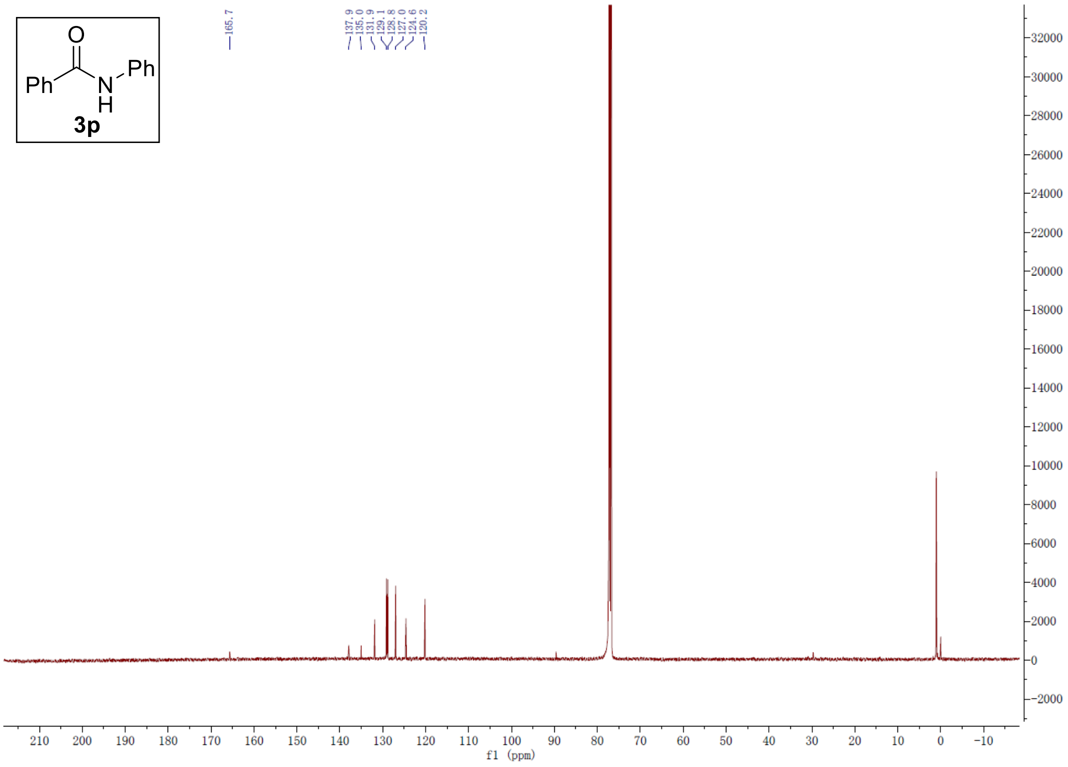

➢ ^{1}H NMR spectrum for **3q**

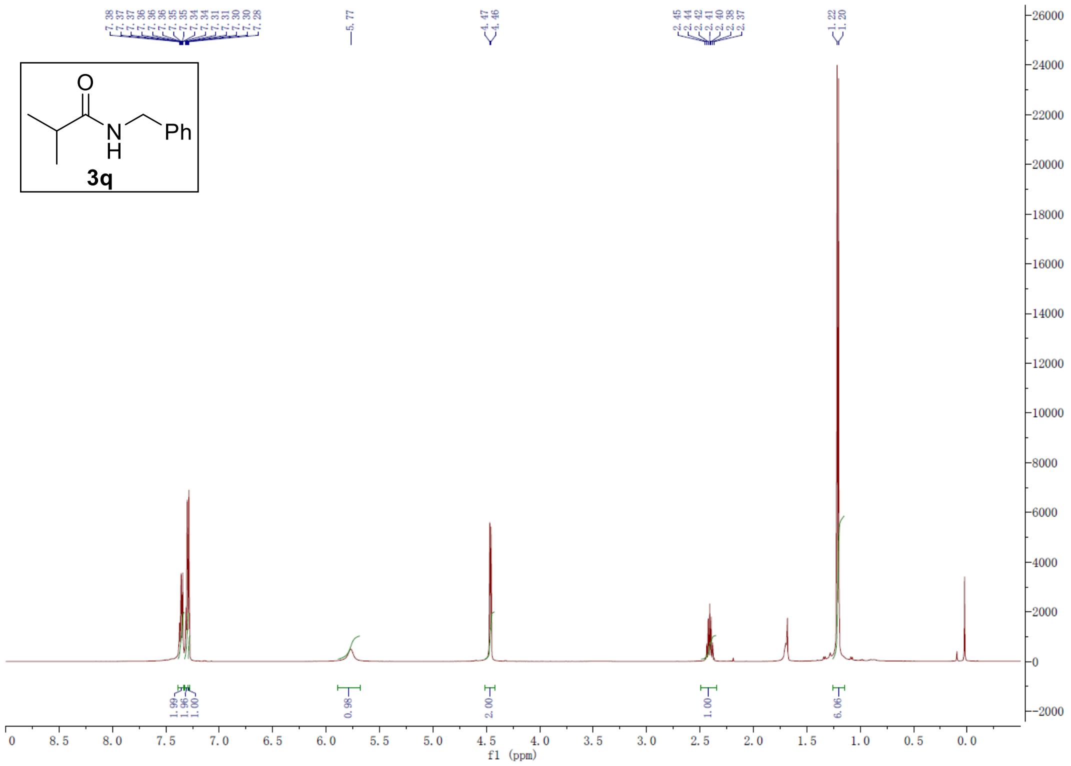

➢ ^{13}C NMR spectrum for **3q**

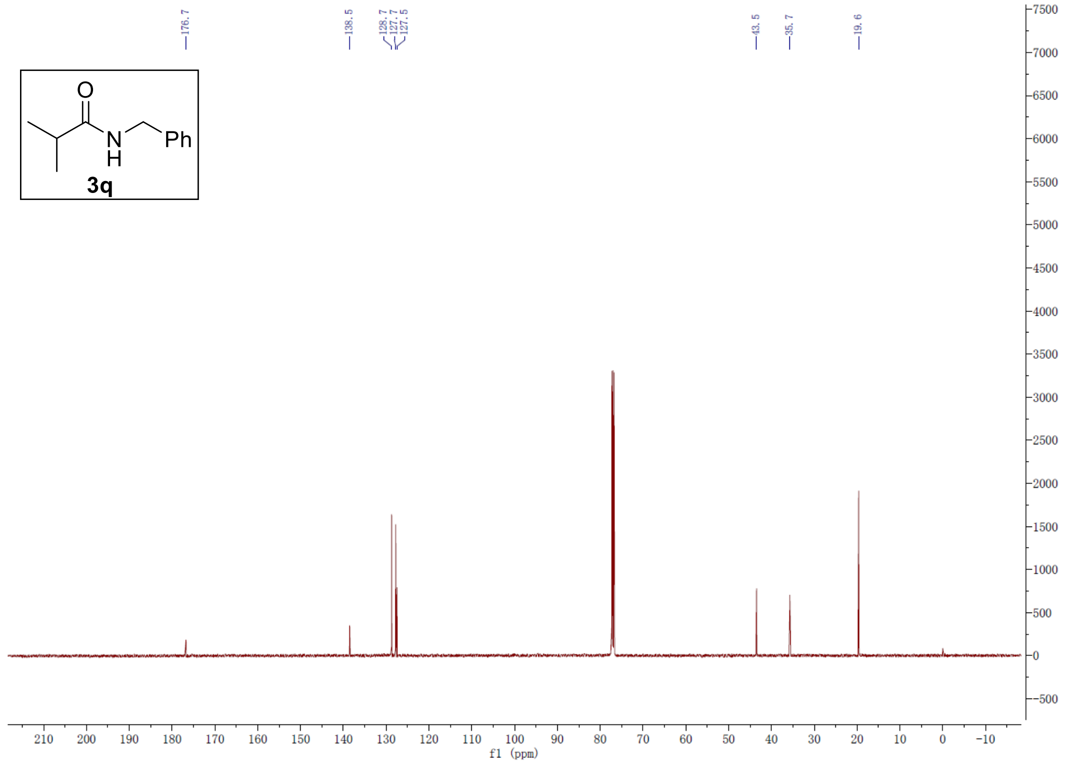

➢ ^{1}H NMR spectrum for **3r**

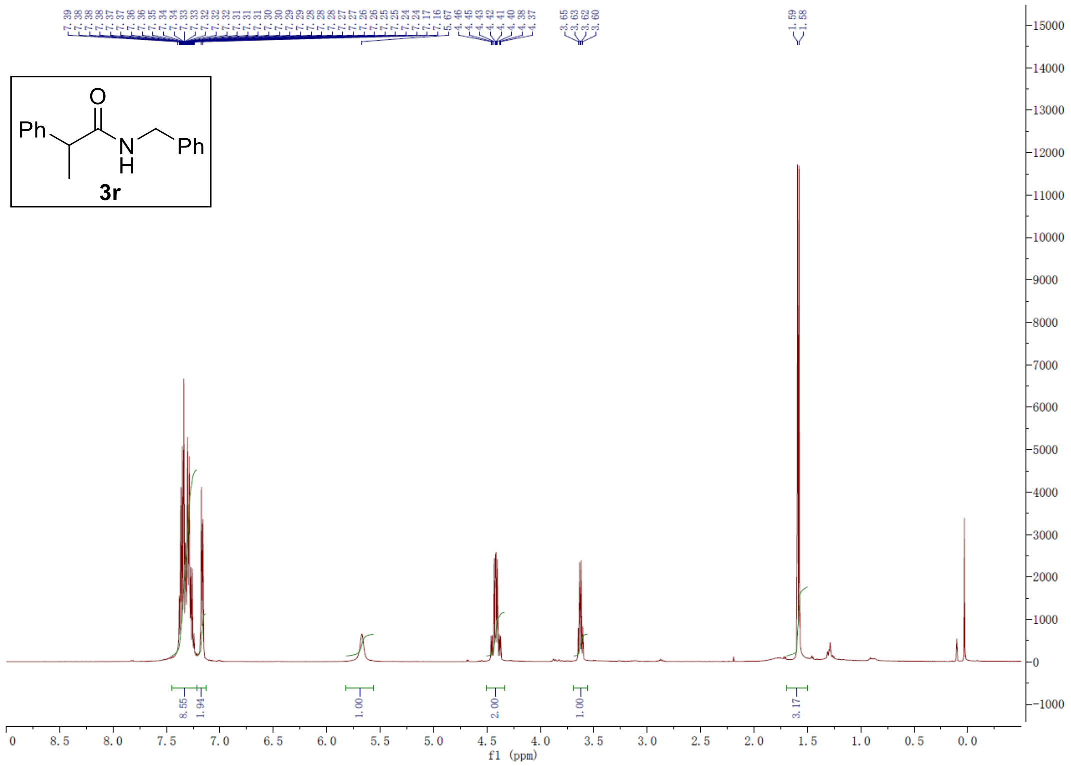

➢ ^{13}C NMR spectrum for **3r**

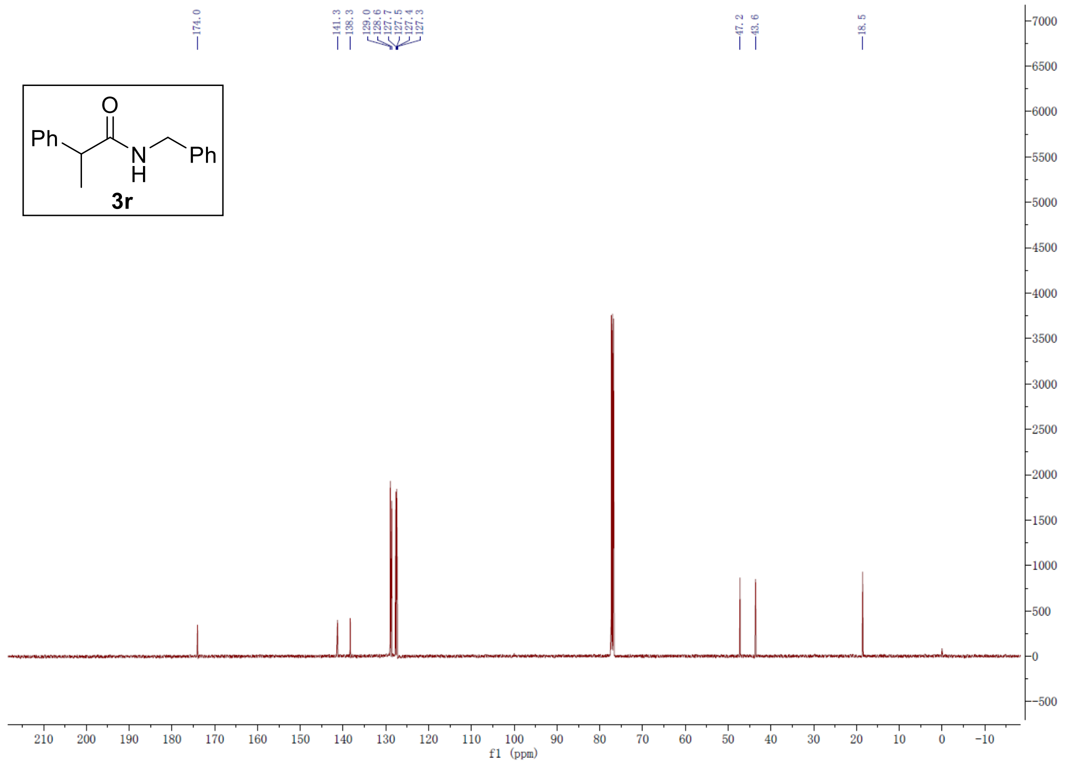

➢ ^{1}H NMR spectrum for **3s**

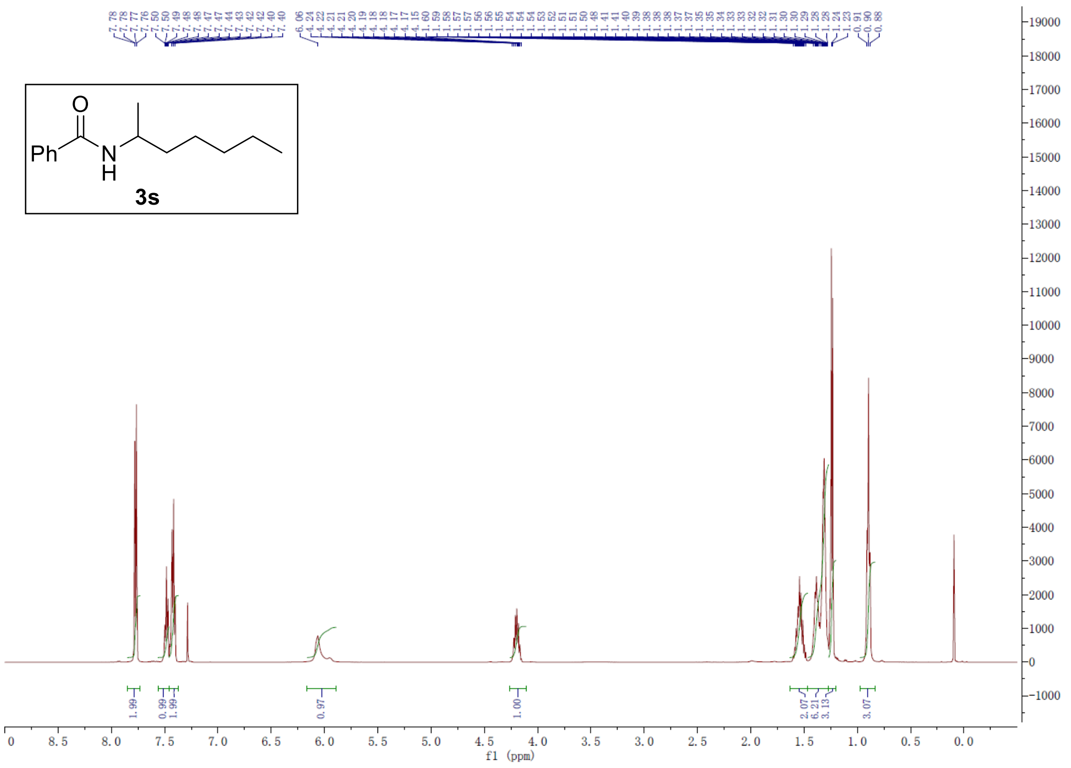

➢ ^{13}C NMR spectrum for **3s**

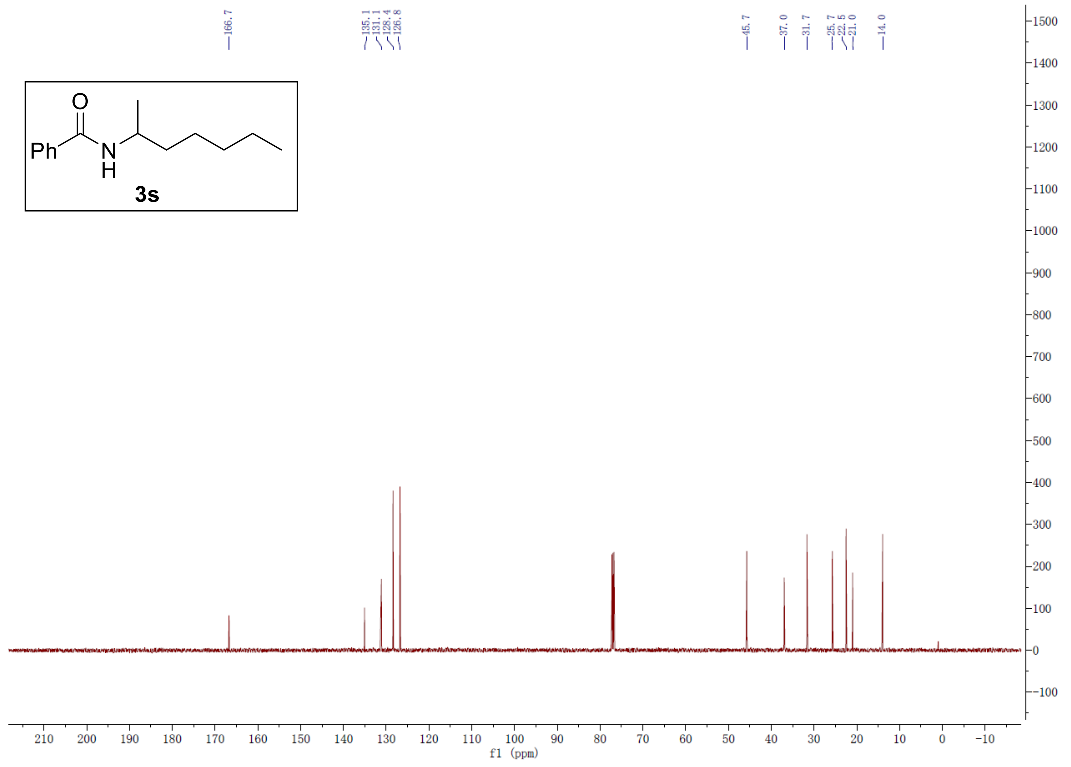

➢ ^{1}H NMR spectrum for **3t**

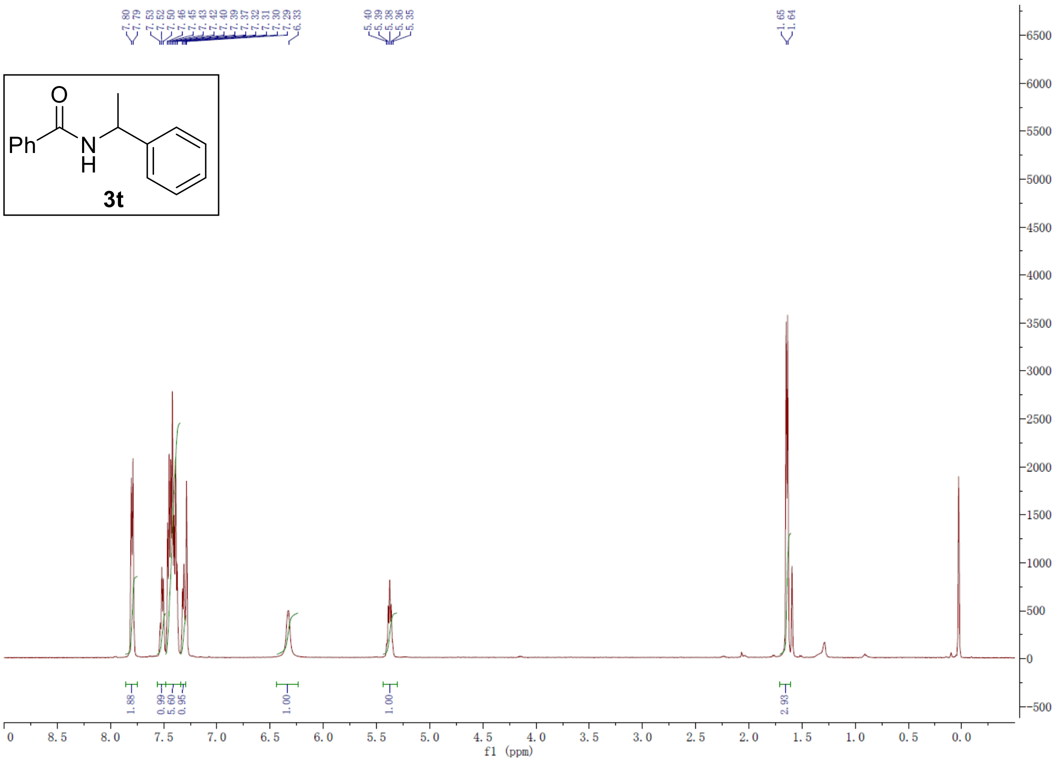

➢ ^{13}C NMR spectrum for **3t**

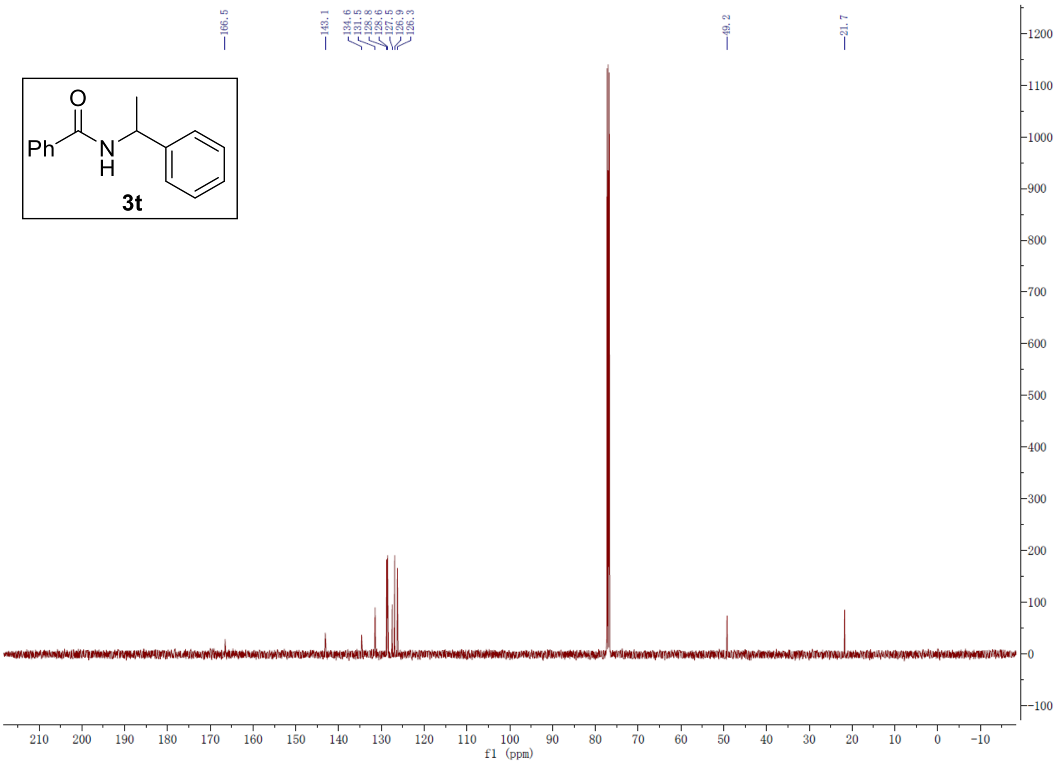

Supplement: Supplementary file 1 [file molecules-23-02413-s001.pdf]
